# Chromatin organization at the nuclear pore favours HIV replication

**DOI:** 10.1038/ncomms7483

**Published:** 2015-03-06

**Authors:** Mickaël Lelek, Nicoletta Casartelli, Danilo Pellin, Ermanno Rizzi, Philippe Souque, Marco Severgnini, Clelia Di Serio, Thomas Fricke, Felipe Diaz-Griffero, Christophe Zimmer, Pierre Charneau, Francesca Di Nunzio

**Affiliations:** 1 Institut Pasteur, Unité Imagerie et Modélisation, CNRS 3691, Paris 75015, France; 2 Institut Pasteur, Unité Virus et Immunité Unité, CNRS 3015, Paris 75015, France; 3 Vita-Salute San Raffaele University Centre for Statistics in the Biomedical Sciences, Milan 20132, Italy; 4 Institute for Biomedical Technologies (ITB), CNR, via F.lli Cervi 93, Segrate 20090, Italy; 5 Institut Pasteur, Unité Virologie Moléculaire et Vaccinologie, CNRS 3569, Paris 75015, France; 6 Department of Microbiology and Immunology, Albert Einstein College of Medicine, Bronx, New York 10461, USA

## Abstract

The molecular mechanisms that allow HIV to integrate into particular sites of the host genome are poorly understood. Here we tested if the nuclear pore complex (NPC) facilitates the targeting of HIV integration by acting on chromatin topology. We show that the integrity of the nuclear side of the NPC, which is mainly composed of Tpr, is not required for HIV nuclear import, but that Nup153 is essential. Depletion of Tpr markedly reduces HIV infectivity, but not the level of integration. HIV integration sites in Tpr-depleted cells are less associated with marks of active genes, consistent with the state of chromatin proximal to the NPC, as analysed by super-resolution microscopy. LEDGF/p75, which promotes viral integration into active genes, stabilizes Tpr at the nuclear periphery and vice versa. Our data support a model in which HIV nuclear import and integration are concerted steps, and where Tpr maintains a chromatin environment favourable for HIV replication.

## Abstract (web summary)

Retroviruses such as HIV integrate into the host genome as an essential step prior to their replication. Here Lelek *et al*. identify nuclear pore complex proteins that are essential for HIV nuclear import and productive integration, and show that the intranuclear protein Tpr influences integration into transcriptionally active chromatin.

## Graphical abstract

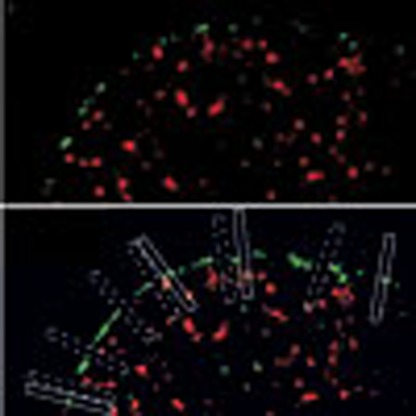

Nuclear pore complexes (NPCs) are stable structures with specific functions in nuclear transport, genome organization, genome stability and gene expression regulation. The interaction between NPCs and specific chromosomal regions, called Nucleoporins (Nups)-associated regions^1^,^2^,^3^,^4^,^5^, indicates that the NPC could have a role in organizing three-dimensional nuclear architecture^6^. The NPC is also a host partner for many viruses that replicate in the nucleus^7^. Lentiviruses, such as HIV-1, usurp the cellular nuclear transport machinery to enter the host nucleus and access the human genome^8^,^9^,^10^,^11^,^12^,^13^,^14^,^15^. The passage of the viral preintegration complex (PIC) through the NPC is an obligated step for the virus to integrate and replicate^16^. The PIC contains the retrotranscribed viral DNA, able to integrate in the human genome through the action of the viral integrase (IN) and host-cell factors, such as LEDGF/p75 (refs 17, 18, 19).

Recently, several studies investigated the mechanistic requirements of Nups^8^,^9^,^10^,^11^,^12^,^13^,^14^,^20^ in the HIV-1 life cycle. The Nups Nup358/RANBP2 and Nup153 have been shown to participate in HIV-1 nuclear import and interact with HIV-1 capsid (CA)^13^,^14^, likely via their FG repeats^9^,^14^,^21^,^22^. These factors could possibly act in concerted or sequential steps. Nup153 as well as Tpr are located at the nuclear basket side^23^. Nup153 is the most dynamic Nup^24^. Its N-terminus domain anchors Nup153 itself and Tpr to the NPC^25^, while the flexible C-terminus domain can be exposed at the cytoplasmic side to enhance the translocation of factors such as Kapβ1^26^ and possibly HIV-1. Tpr forms filamentous structures that extend into the nuclear interior^27^,^28^,^29^ and contrary to Nup153 its mobility is unknown.

In this study we unravel the distinct roles of Nup153 and Tpr in HIV-1 infection. We observed that Nup153 participates in HIV-1 nuclear import independently of the integrity of the nuclear basket. Tpr has a fundamental role in chromatin organization, by excluding heterochromatin from the vicinity of NPCs and seems to recruit hypertranscribed genes near the NPC^1^,^30^. We hypothesized that HIV-1 could usurp this role of Tpr to promote its replication. Following this hypothesis, the NPC could create a chromatin topology and transcriptional environment favourable for HIV-1 replication. We tested this hypothesis in Tpr knockdown (KD) cells using a combination of assays, including super-resolution microscopy and pyrosequencing. Our data shows a critical role of the nuclear basket in HIV-1 replication, a connection between NPC and chromatin modifications associated with active genes and highlights the mechanistic requirements of NPCs in viral gene expression. This study contributes to our understanding of viral silencing, with possible implications for HIV-1 persistence.

## Results

### Critical and distinct roles of Nup153 and Tpr in HIV infection

**Figure 1:**
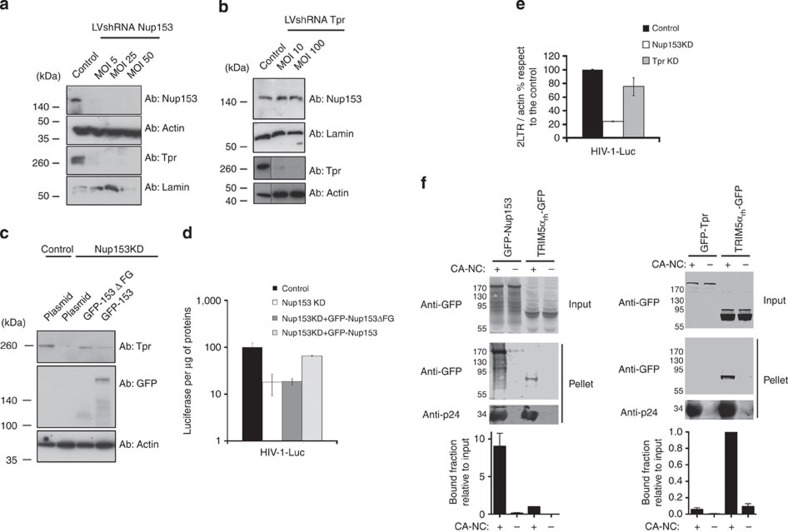
Depletion of Nup153 and Tpr followed by reconstitution of the nuclear basket by complementation and *in vitro* CA–NC binding assay. (**a**,**b**) We used the pTrip.GFP.H1shRNA vector to knockdown (KD) the expression of Nup153 and Tpr in HeLa P4CCR5 cells^13^,^14^. Viral particles produced using the pTrip.GFP.H1shRNA vector containing the specific shRNA against Nup153 and Tpr were used to transduce HeLa P4CCR5 at the indicated multiplicity of infection (MOI). Control cells were transduced with the empty pTrip.GFP.H1shRNA vector. The efficiency of the KD was monitored by western blotting using antibodies against the endogenous Nup153 proteins and Tpr. As a loading control, samples were also blotted using antibodies against actin and lamin A/C. (**c**) Nup153-depleted cells at MOI 50 were complemented with a plasmid, pC2GFP, GFP-Nup153w/o FG and GFP-Nup153. The efficiency of the complementation was monitored by western blotting using antibodies against the endogenous Tpr, GFP and actin. (**d**, **e**) Nup153 and Tpr -depleted cells were challenged with HIV-1 containing luciferase as a reporter of infection. HIV-1-Luc was normalized by p24 as described in Methods. Infectivity was determined 48 h p.i. by measuring luciferase activity normalized to the amount of protein. 24 h p.i. total genomic DNA from infected cells was used to measure 2LTR circles by real-time PCR normalized to actin. The percentage of 2LTR circles with respect to the control is shown in the histograms of Nup153 and Tpr-depleted cells at MOI 50 and 100, respectively. The s.d. of three independent experiments are shown. (**f**) The binding of GFP-Nup153 and GFP-Tpr proteins to *in vitro* assembled HIV-1 capsid–nucleocapsid (CA–NC) complexes was performed by transfecting human 293T cells with plasmids expressing GFP-Nup153, GFP-Tpr, GFP and Trim5α_rh_-GFP. The assay is described in Methods. Quantification of BOUND versus INPUT fractions of three independent experiments was plotted with their respective standard deviations on the lower panels.

**Figure 2:**
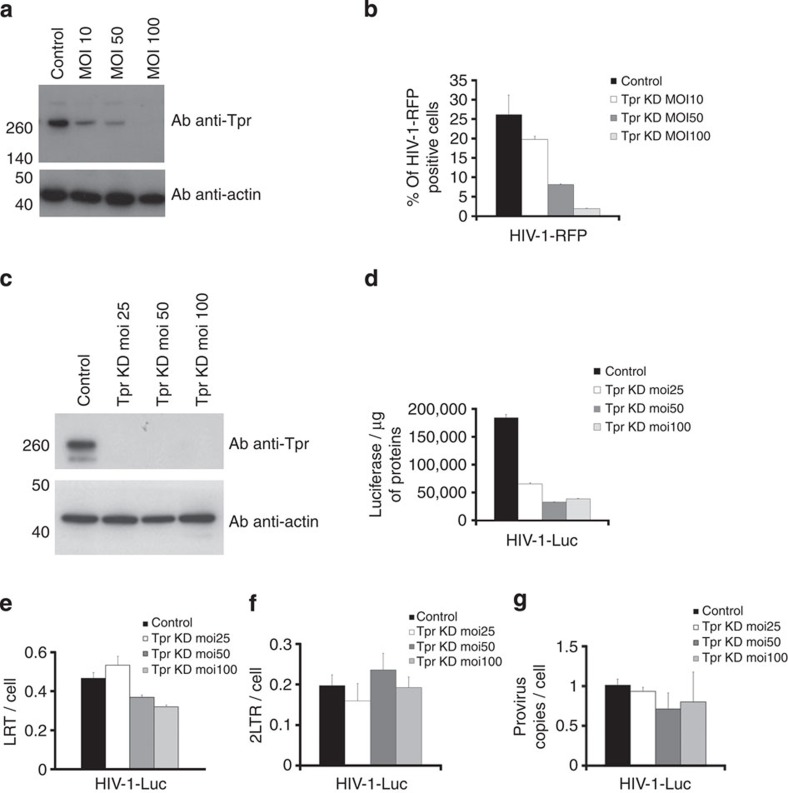
Involvement of Tpr in HIV-1 replication. (**a**) HeLa P4CCR5 cells were depleted for Tpr using different doses of LVshRNA against Tpr and (**b**) infectivity was measured 48 h p.i. by flow cytometry (**c**) Jurkat cells were depleted for Tpr using different doses of LVshRNA against Tpr, as shown in the western blot and (**d**) infectivity was measured 48 h p.i. by luciferase activity normalized to the amount of protein. (**e**,**f**,**g**) Viral late reverse transcription (LRT) levels were analysed at 7 h p.i., nuclear import and integration were analysed at 24h p.i. by 2LTRs and Alu-PCR, respectively. Infections carried out in the presence of Nevirapine at 5 μM led to undetectable levels of LRT, 2LTR circles and Alu-PCR. Similar results were obtained in three independent experiments and s.d. are shown.

Tpr is the main component of the nuclear basket and docks at the NPC exclusively by a short segment located at its amino-terminal domain^25^. This segment strongly interacts with the amino-terminal region of Nup153 (aa 228–439), which is also essential for anchoring Nup153 to the NPC itself^31^. Tpr and Nup153 are both located at the nuclear side of NPCs, possibly exercising divergent and complementary functions. To investigate the individual role of these Nups, we silenced their expression in human HeLa P4-CCR5 cells^32^, which express ß-galactosidase under the control of the HIV-1 long terminal repeat (LTR) promoter transactivated by the viral tat protein, using lentiviral particles expressing specific shRNAs (LVshRNA) with and without GFP as reporter. Three days after transduction of HeLa P4-CCR5 at the indicated multiplicities of infection (MOI), expression of Nup153 and Tpr was monitored by western blotting (Figs 1a,b and 2a). Next, we challenged Nup153 and Tpr-depleted cells with HIV-1ΔEnv pseudotyped with vesicular stomatitis virus G protein (VSV-G) expressing the luciferase (Luc; HIV-1-Luc) or RFP (HIV-1-RFP) measured 48 h post infection (p.i.) by luminescence assay or flow cytometry (Figs 1d and 2b). We observed a defect in infectivity of up to 10-fold in both, Nup153 and Tpr-depleted cells (Figs 1d and 2b). These results suggest that Nup153 and Tpr are both involved in HIV-1 infection.

However, distinguishing the specific roles of Nup 153 and Tpr is challenging because of their above-mentioned tight structural connection^25^. Indeed, we confirmed that Nup153- depleted cells are also depleted for Tpr^25^, making it difficult to properly attribute the detected infectivity phenotype exclusively to either Nup (Fig. 1a). By contrast, Tpr depletion did not affect the level of Nup153 (ref. 33) (Fig. 1b). In order to investigate the role of Nup153 in presence of an intact nuclear basket, we performed a complementation assay. We complemented Nup153-depleted HeLa P4-CCR5 cells with GFP-Nup153 or with the deletion mutant of Nup153 that lost the entire C-terminus region containing FG repeats (aa 896–1475; GFP-Nup153ΔFG). Both complementations restored the expression of Tpr to the level of control cells (Fig. 1c). This model allows to specifically study the role of the C-terminus region of Nup153 in HIV-1 infection in a quasi—wild-type nuclear basket. We challenged the complemented cells with HIV-1-Luc. Complementation with GFP-Nup153ΔFG restored the expression of a truncated Nup153 able to bind the NPC because it contains the N-terminus domain that anchors itself and the Tpr fibrils to the NPC^25^,^31^ (Fig. 1c), but did not rescue viral infectivity (Fig. 1d). Complementation with the full length GFP-Nup153 not only recovered the expression of both Nup153 and Tpr (Fig. 1c), but also rescued near wild-type levels of infectivity (Fig. 1d). These results show that the reduction of HIV-1 infection in Nup153 KD cells is exclusively due to the loss of the C-terminus domain of Nup153 and not to the absence of Tpr (Fig. 1c,d).

Nup153 contains FG repeats, which interact with *in vitro* assembled cores and intervene in HIV-1 nuclear import (Fig. 1e,f). We confirmed the latter^9^,^10^,^13^,^14^ by analysing 2 LTR circles, a common measure of nuclear import^9^,^10^,^11^,^12^,^13^,^14^,^34^,^35^,^36^. This assay also showed that Tpr is not involved in HIV-1 nuclear import (Fig. 1e). We also asked whether Tpr, which has no FG repeats, can interact with *in vitro* assembled cores, which mimic the CA lattice of mature viral cores^37^. This was not the case (Fig. 1f). Taken together, these results show how Nup153 acts independently of Tpr in HIV nuclear import.

### Role of Tpr fibrils in HIV-1 replication

We further explored the functional role of the nuclear basket in HIV-1 infection using Tpr-depleted cells. Tpr expression was silenced in human HeLa P4-CCR5 cells^32^ and in T-cell lymphoblast-like cells (Jurkat) using different doses of LVshRNA (Fig. 2a,c). Next, we challenged Tpr-depleted cells with a single round virus (Fig. 2b,d) and measured infectivity as above. In HeLa P4CCR5 we observed a correlation between the level of expression of Tpr and the infectivity defect, showing a specificity of Tpr for HIV-1 infection (Fig. 2a). In Jurkat cells we achieved strong KD of Tpr expression already at MOI 25 (Fig. 2c). Next, we aimed to determine the step of the HIV-1 life cycle that requires Tpr. We first measured the DNA synthesis at 7 h p.i. (Fig. 2e) and the production of 2LTRs in the Tpr-depleted Jurkat cell line which showed the strongest silencing, using real-time PCR at 24 h p.i. (Fig. 2f). Depletion of Tpr did not affect HIV-1-Luc reverse transcription and production of 2LTR circles during infection compared with control cells (Figs 1e and 2e,f), suggesting that Tpr is not involved in HIV-1 DNA synthesis and nuclear import. In parallel, we performed Alu-PCR at 24 h p.i. to assess the integration of HIV-1 viral DNA into the host chromatin (Fig. 2g). We observed no significant reduction of integrated HIV-1 proviruses in Tpr-depleted cells compared to infected control cells even at LV-shRNA MOI of 100 (Fig. 2d,g). Overall, these results show that the depletion of Tpr strongly reduces the ability of HIV-1 to infect cells (Fig. 2b,d) without altering reverse transcription, nuclear import or the level of integration (Fig. 2e–g).

**Figure 3:**
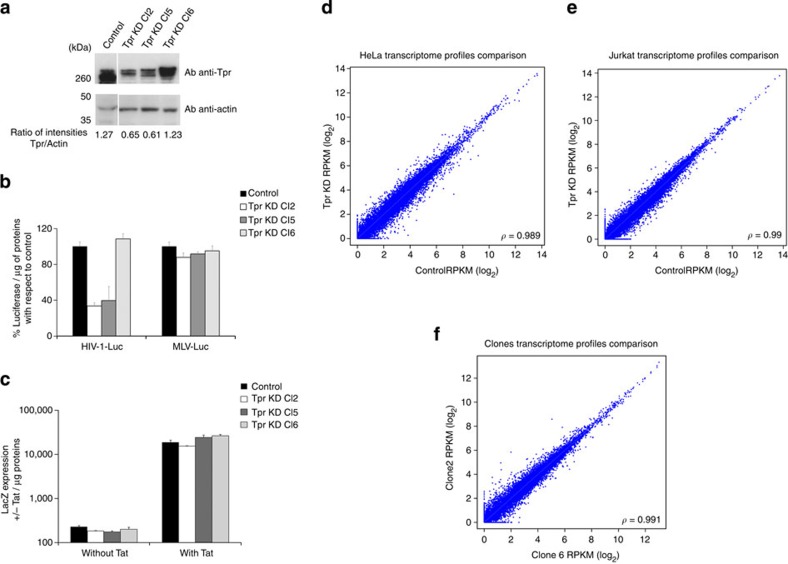
Tpr is specifically involved in HIV-1 infection. (**a**) Three out of ~10 final clones obtained by limiting dilution were selected for the expression of Tpr by western blotting and (**b**) MLV-Luc and HIV-1-Luc were used in parallel to infect the control clone (obtained with an empty LV) and clones 2, 5 and 6. (**c**) Tat was transfected in all clones to evaluate LacZ expression by β-galactosidase assay normalized to the amount of protein. Clones were thawed and frozen multiple times and yielded similar results (s.d. are shown). (**d**–**f**) Transcriptome profiles comparison: scatter plots show RPKM values observed in ~36,000 annotated in control sample (*x* axis) and Tpr KD (*y* axis). Scatter plots of Pearson correlation calculated using the RNA-seq log2 RPKM values of Tpr depleted versus control for (**d**) HeLa cells, (**e**) Jurkat cells and (**f**) clones.

To investigate the role of Tpr in longer testing conditions, we selected stable HeLa P4-CCR5 clones by limiting dilution. We analysed by western blot multiple clones with different level of expression of Tpr normalized by actin (Fig. 3a) and evaluated their ability to be infected with HIV-1-Luc (Fig. 3b). We observed the strongest phenotype in HIV-1 infection in clones 2 and 5 that stably expressed half of the quantity of Tpr detected in the wild-type clone or in Tpr KD clone 6 (Fig. 3a). To test the specificity of Tpr in HIV-1 infection, we infected these clones in parallel with HIV-1-Luc and MLV (Murine Leukaemia Virus)-Luc. MLV requires mitosis to access the nucleus. Importantly, the clones proliferated similarly (Supplementary Fig. 1). After infection we detected a defect of Luc expression only in the HIV-1 infected clones that underexpressed Tpr (Fig. 3a,b).

**Figure 4:**
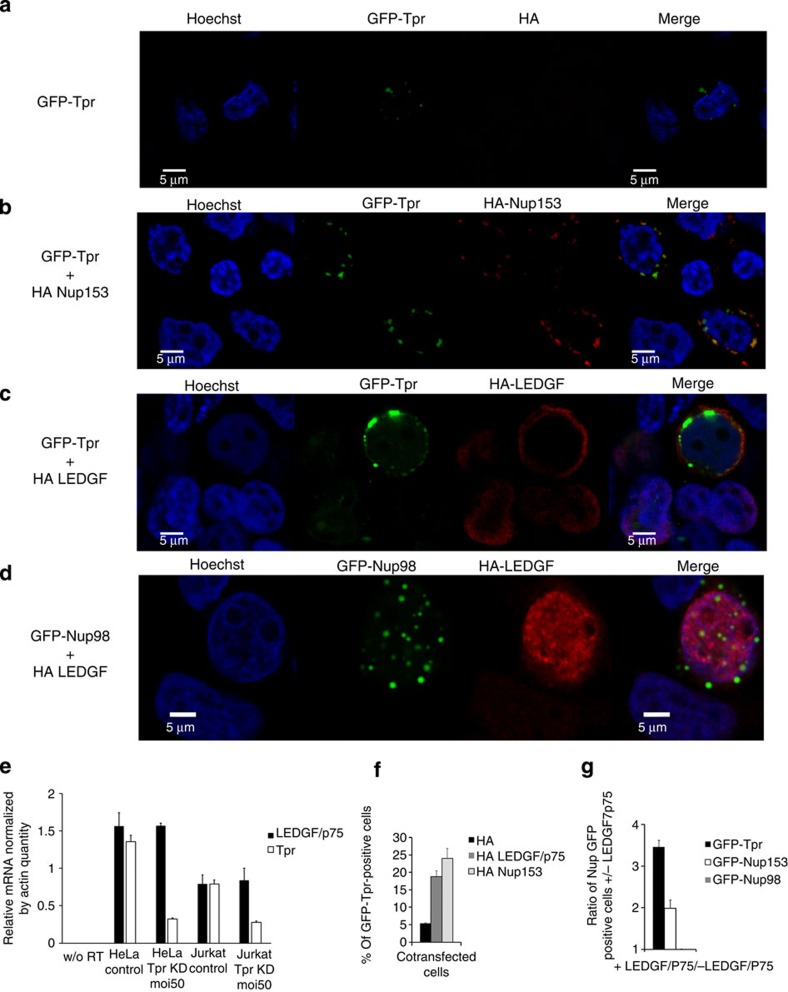
Increase of intensity of Tpr at the nuclear periphery by LEDGF/p75 overexpression and vice versa. The 293 T cells were cotransfected with GFP-Tpr and (**a**) HA alone or (**b**) HA-Nup153 or (**c**) HA-LEDGF/p75. (**d**) As control we cotransfected GFP-Nup98 with HA-LEDGF/p75. HA fused proteins were detected using a monoclonal anti-HA antibody and a secondary antibody conjugated with Cy3. (**e**) RT–PCR, mean of ratio between relative mRNA of PSIP1 and of Tpr genes by actin in Tpr depleted versus control HeLa and Jurkat cells is shown in the histogram. (**f**) Flow cytometry analysis show the percentage of GFP-Tpr-positive cells when cotransfected with HA, HA-LEDGF/p75 and HA-Nup153. (**g**) Relative increase of Nup GFP-positive cells on overexpression of LEDGF/p75 relative to the endogenous level of LEDGF/p75 based on flow cytometry data. S.d. of three independent experiments are shown.

We next asked if this Luc expression defect reflected a global change of gene expression rather than a specific reduction of HIV-1 expression. To test this, we transfected a plasmid expressing Tat to transactivate HIV-1 LTR promoter in all clones and compared the expression of LacZ by β-Galactosidase assay in presence or absence of Tat. We could not detect any difference in LacZ expression between clones (Fig. 3c), despite different expression levels of HIV-1 reporter genes (Fig. 3b). We tested if this could result from a role of Tpr in mRNA export^38^ by quantifying the ratio between the cytoplasmic RNA versus the nuclear RNA fractions. The cytoplasmic/nuclear RNA fractions in HeLa cells and in clones were not changed by Tpr depletion, whereas this ratio was reduced on Nup214 depletion, in agreement with previous data^14^ (Supplementary Fig. 2). To investigate if Tpr depletion affects global gene expression, we compared the transcription level of 36,064 genes on Tpr-depleted HeLa cells versus control cells; Tpr-depleted Jurkat cells versus control cells; and clone 2 (low Tpr expression) versus clone 6 (high Tpr expression). To measure the overall gene expression similarity between Tpr-depleted cells and controls, we calculated the RPKM (reads per kilobase of transcript per million mapped reads) values as an estimation of transcript abundance and evaluated the Pearson correlation for each comparison. For all three pairs, the expression profiles were very similar (Pearson correlation coefficients close to 1; Fig. 3d–f). In addition, for HeLa and Jurkat cells, we used RT–PCR to compare the mRNA levels of the following specific genes: Actin, GAPDH, Trim24, SNX27, PSIP1 and Tpr. We again observed similar mRNA levels between Tpr KD and control cells for all genes except Tpr (Fig. 4e; Supplementary Fig. 3). Altogether, these data support a specific role of Tpr in HIV-1 gene expression without global alteration of gene expression.

### Role of Tpr in HIV-1 integration sites distribution

**Figure 5:**
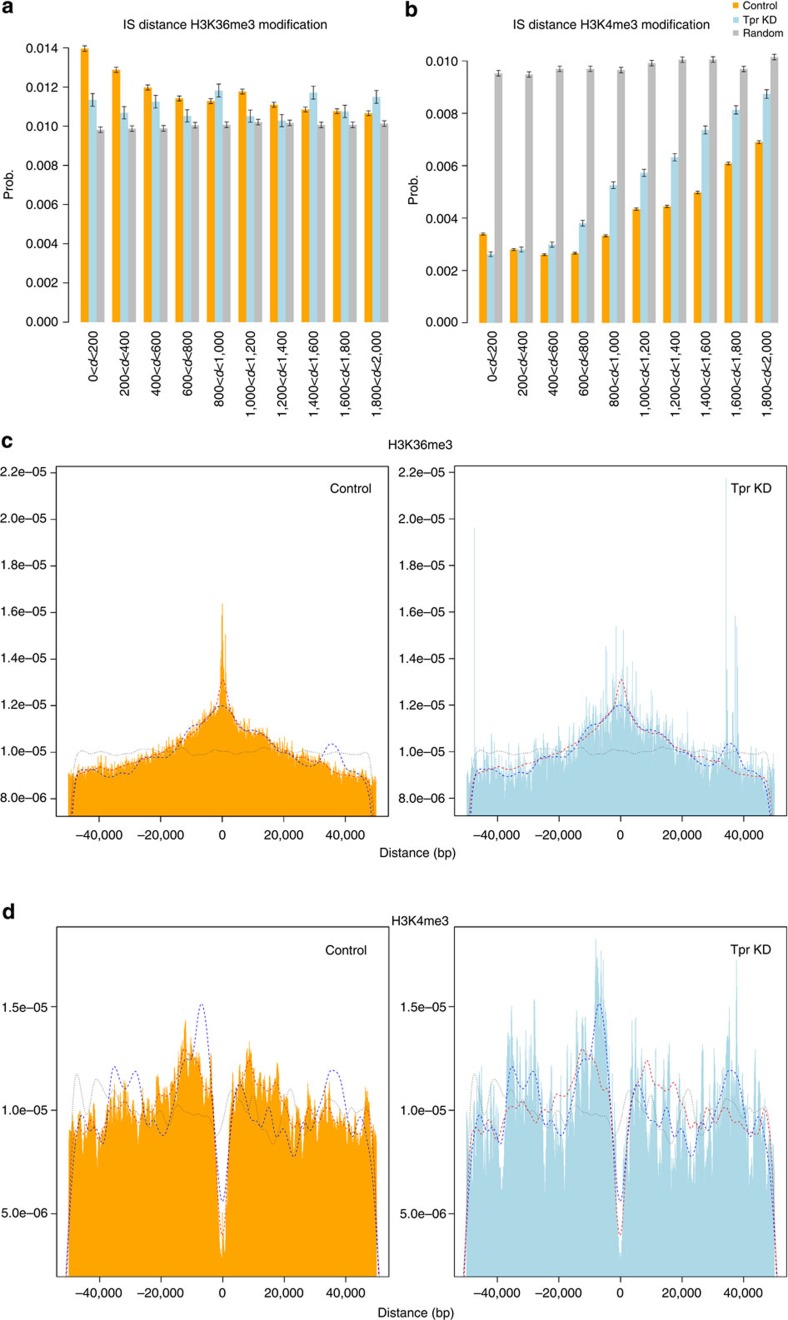
Short-range association between integration sites and genomic features. (**a**, **b**) Histograms show the distribution of absolute genomic distances to specific features of HIV-1 integration sites, with bin size of 200. The genomic features are Histone methylation H3K36me3 and H3K4me3 in a 2 kb window centred on integration sites. The distributions are shown for control cells (orange bars), Tpr KD cells (light blue bars) and as predicted for random integration (grey bars). (**c**) Frequency of ISs from histone methylation H3K36me3 and (**d**) H3K4me3, as function of genomic distance, plotted with bins of 100 bp in a ±50 kb window centred on integration sites in control cells (left, orange) and Tpr KD cells (right, blue). Dashed curves correspond to kernel density estimations of the distance distribution for control cells (red), Tpr KD (blue) and random integration (grey).

The absence of Tpr induces the presence of more condensed chromatin near the nuclear basket, influencing chromatin organization at the nuclear periphery^30^. We hypothesized that depletion of Tpr may affect HIV-1 integration site selection and efficiency by disrupting the link between the nuclear basket and the underlying chromatin, whose state might be important to allow HIV-1 replication. To test this hypothesis, we isolated HIV-1-Luc integration sites from Tpr-depleted Jurkat cells challenged with a single round virus at a MOI of 50 (Fig. 2d). As a control, we challenged Jurkat cells containing the shRNA empty vector with HIV-1-Luc. After digestion and ligation, we amplified the integration sites by nested PCR using as a template the ligated DNA. We used primers annealing to the HIV-1 U3 region, which is absent in shRNA expressing self-inactivated (ΔU3) lentiviral vectors, to selectively amplify HIV-1-Luc integration sites^13^ and performed 454-pyrosequencing. Control and Tpr-depleted Jurkat cell libraries generated 106,100 and 144,920 raw reads, respectively. These reads were filtered to remove duplicates, short sequences and LM–PCR artefacts, using a previously described pipeline^13^. After removal of provirus and amplification linker sequences, a total of 9,509 and 4,857 reads was obtained for each library in control and Tpr-depleted Jurkat cells, respectively, and mapped to the human genome (UCSC release hg19). All UCSC known genes with transcription start site (TSS) at ±50 kb from an integration were considered. Integrations were annotated as (i) TSS-proximal if mapping inside a ±2.5 kb interval around a TSS of known genes, (ii) intragenic if mapping inside exons or introns of a known gene and (iii) intergenic when upstream or downstream of known genes. Supplementary Table 1 reports the frequencies of these annotations for control Jurkat cells, Tpr KD Jurkat cells and the frequencies expected for random integration. We also measured the proportion of integrations in regions containing genomic features associated with open chromatin, based on available data sets (UCSC and ENCODE Jurkat; Fig. 5a–d; Supplementary Fig. 4). Compared with both, control cells and random integrations, Tpr-depleted cells exhibited less integrations mapping to regions enriched in DNase I hypersensitive (HS) sites or histone modification H3K36me3, which are associated with actively transcribed genes (Fig. 5a,c; Supplementary Fig. 4a,b). However, in both Tpr KD and control Jurkat cells, HIV-1 integrations are disfavoured in regions bearing histone modification H3K4me3, which is associated to regulatory regions, compared with random integrations (Fig. 5b,d).

In summary, these data show that Tpr depletion reduces the association of HIV-1 integration sites with marks of open chromatin.

### TPR role in chromatin topology underneath the NPC

**Figure 6:**
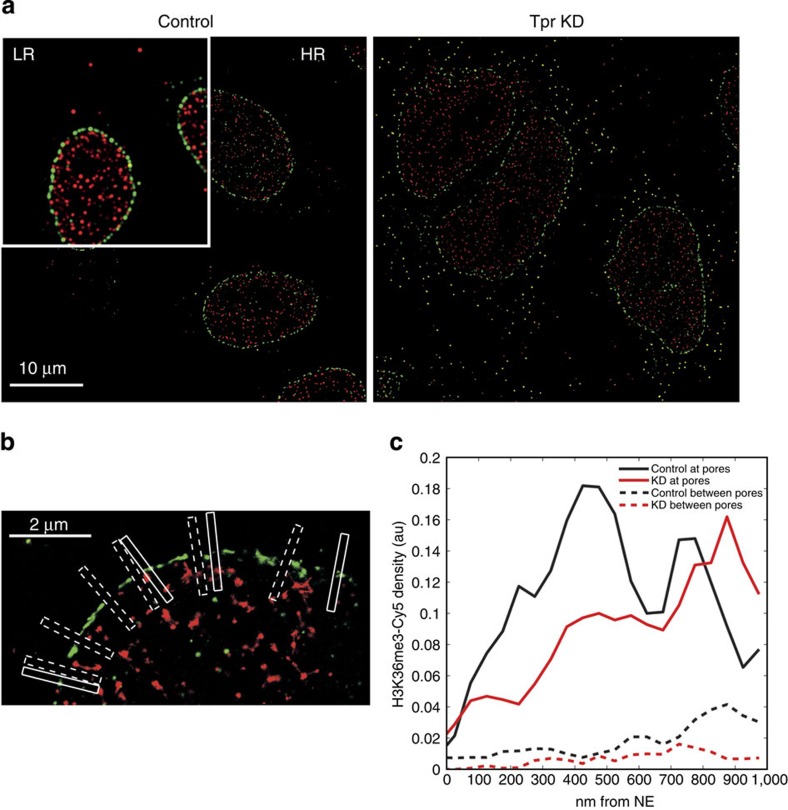
Super-resolution imaging of active chromatin underneath NPCs in control and Tpr depleted HeLa cells. (**a**) STORM image (visualized as smoothed histogram of computed positions) of Nup153-A568 (green) and H3K36me3-Cy5 (red) in control cells (left) and Tpr KD cells (right). The top left corner of the figure shows the low resolution image that would have been obtained with a conventional (diffraction limited) microscope to appreciate the gain in resolution. The yellow spots outside of the nuclei are multicolour fluorescent beads used to correct sample drift and chromatic shifts. (**b**) STORM image (visualized as scatter plots of computed positions): zoom on a part of left image in panel **a** showing rectangles oriented across the NE and passing either through NPCs (solid boxes) or in between neighbouring NPCs (dashed boxes). The positions of boxes are defined manually based on the green (NPC or NE) channel only. These boxes are used to quantify the distribution of H3K36me3 in panel c. (**c**) Density profiles of H3K36me3 as function of distance from the NE, computed from the rectangular boxes as shown in **b**. Solid curves correspond to boxes passing through NPCs, dashed curves to boxes passing in between neighbouring NPCs. Black curves correspond to control cells, red curves to Tpr KD HeLa P4CCR5 cells. The profiles were computed as described in Methods from 122 NPCs and 305 positions between NPCs in Tpr KD cells versus 140 NPCs and 265 positions between NPCs in control cells; au, arbitrary units.

We next wished to determine if the observed change in integration site preferences on Tpr depletion could arise from a lack of active chromatin near NPCs. For this purpose, we aimed to evaluate the density of the histone modification H3K36me3, which is associated with both HIV-1 integration sites and actively transcribed genes^39^, near NPCs in Tpr-depleted versus control HeLa cells. Because the size of individual NPCs (~120 nm)^6^ falls below the resolution limit of conventional light microscopy (~200–300 nm), we used a super-resolution imaging technique, STochastic Optical Reconstruction Microscopy (STORM)^40^ with two colours to simultaneously visualize NPCs and H3K36me3 (Fig. 6; Methods). For labelling, we used secondary antibodies coupled to the synthetic dyes Alexa 568 (A568) and Cy5.

As an initial control we performed dual-colour STORM of two Nups: Nup153-A568 and Nup214-Cy5. Images were obtained in a focal plane near the nuclear equator. In the reconstructed STORM images, the Nup214 signal at the nuclear envelope was close to, but mostly distinct from the Nup153 signal, and located outside of it, with a median distance between the intensity peaks at individual pores of ~130 nm (Supplementary Fig. 5). Given our estimated resolution of ~30 nm (computed using the method of ref. 41), this distance is in good agreement with the location of these two Nups as previously determined by cryoelectron microscopy^42^, confirming the quality of these super-resolution images (see Methods). We then applied dual-colour STORM to image Nup153-A568 together with H3K36me3-Cy5 (Fig. 6a). Next, we quantified the density of H3K36me3 signal along 50 nm wide stripes perpendicular to the NE and passing either through individual NPCs or between neighbouring NPCs (Fig. 6b; see Methods). This was done for a total of 140 NPCs and 265 positions between NPCs in control cells, and for 122 NPCs and 305 positions between NPCs in Tpr KD cells. Results are shown in Fig. 6c. In control cells the density of H3K36me3 was much larger in stripes passing through NPCs than in between NPCs, with a peak density at ~500 nm from the NPC position. This accumulation of an active chromatin mark underneath NPCs is in agreement with the previously reported lack of heterochromatin at these locations relative to the rest of the nuclear periphery^30^. In Tpr-depleted cells the density of H3K36me3 was also more abundant underneath NPCs than in between. However, we observed a reduction in H3K36me3 density at distances of ~100–500 nm underneath NPCs compared with control cells (Fig. 6c).This decreased density of an active chromatin mark is consistent with previous electron microscopy observations showing that heterochromatin exclusion from the vicinity of NPCs depends on Tpr^30^. This observation together with the weaker presence of H3K36me3 at HIV-1 integration sites in Tpr KD cells (Fig. 5a,c), is consistent with a preferential integration of HIV-1 into chromatin near the nuclear periphery^43^. Our results promotes the nuclear pore basket as critical cellular factor to ensure a favourable chromatin environment, such as the presence of H3K36me3, underneath NPCs for viral replication. This could help to explain the low level of expression of integrated proviruses observed in Tpr-depleted cells (Fig. 2b,d).

### Tpr promotes the relocalization of LEDGF/p75 near the NE

LEDGF/p75, the only cellular factor known to drive the PIC into active chromatin regions^44^, is known to directly bind H3K36me3 (ref. 45), which we just showed to be located near the nuclear pore basket (Fig. 6). To investigate a potential link between the nuclear basket and LEDGF/p75, we asked if Tpr has a role in LEDGF/p75 nuclear localization. We cotransfected HA-LEDGF/p75 with GFP-Tpr in 293T cells and used multicolour confocal microscopy and image analysis to evaluate Tpr and LEDGF/p75 localization with respect to the NE (Methods; Fig. 4c; Supplementary Fig. 6c,e). We observed an increase of GFP-Tpr fluorescence near the NE similar to that obtained by overexpression of Nup153 (ref. 25), suggesting that LEDGF/p75 stabilizes Tpr underneath the NPC. When Nup153 is overexpressed, Tpr seems more stabilized at the NE, presumably because there are more anchoring sites^25^ (Fig. 4b). We did not observe this result when we cotransfected GFP-Tpr with an HA empty vector (Fig. 4a). Interestingly, we also observed a relocalization of LEDGF/p75 from the centre towards the nuclear periphery on overexpression of GFP-Tpr or GFP-Nup153, but not of GFP-Nup98 (all three Nups are located at the nuclear side of the NPC; Fig. 4b–d). The nuclear localization of the GFP-fused Nups was consistent with other studies^24^,^46^,^47^. We also confirmed these results by flow cytometry (Fig. 4f,g). The confocal microscopy data also showed colocalization between IN_Flag_ and LEDGF/p75 (refs 48, 49) when displaced near the NE by Tpr overexpression, suggesting the intact functionality of this IN cofactor^50^,^51^ (Supplementary Fig. 6e). Therefore, contrary to LEDGF/p75, HIV-1-IN does not change location on Tpr overexpression (Supplementary Fig. 6g,h). We observed an ~four- and ~twofold increase in density of the western blot band of LEDGF/p75 when cotransfected with GFP-Tpr and GFP-Nup153, respectively, compared with cotransfection with other factors (Nup98 or GFP). We observed similar results independently of the tag fused to LEDGF (Supplementary Fig. 6i). Given that Tpr does not affect global gene expression (Fig. 3d–f), nor the expression of the specific gene PSIP1, which codes for LEDGF/p75 (Fig. 4e), these data support the possibility that Tpr and LEDGF/p75 are part of a complex, which increases the stability of both proteins. A similar role of LEDGF/p75 has been shown when it binds to HIV-1 IN and thereby stabilizes this viral factor^48^,^50^. Our microscopy data (Fig. 4a-d; and Supplementary Fig. 6b–f) suggest that Nup153 acts indirectly by anchoring Tpr to the NPC, while Tpr may have a more active role on LEDGF/p75 and HIV-1 infection.

## Discussion

Despite a large number of studies, the role of the nuclear basket in HIV-1 infection remains elusive. A major difficulty in determining the role of individual Nups is their structural association, whereby many Nups act as scaffold for others. Our data provide direct evidence of the exclusive involvement of Nup153 and Tpr in distinct and complementary steps of the HIV-1 life cycle. Previously, we showed the interaction of Nup153 with *in vitro* assembled HIV-1 cores^13^. The target region for this binding was later found to be the FG repeats located at the C-terminus of Nup153 (ref. 9). Here we showed *in vivo* that the role of Nup153 in HIV-1 nuclear import is unrelated to the integrity of the nuclear basket (Fig. 1c,d). The N-terminus domain of Nup153 is responsible for recruiting itself and Tpr^25^ to the NPC, therefore, in Nup153-depleted cells the integrity of the nuclear basket is lost. By complementation assay we artificially reconstitute the nuclear basket on Nup153-depleted cells, showing that the flexible C-terminus domain of Nup153, is required for HIV-1 nuclear import (Fig. 1c–e). We observed that contrary to Nup153, Tpr, which has no FG repeats, does not interact with *in vitro* assembled cores (Fig. 1f).

Tpr intervenes in several cellular mechanisms, including the mitotic spindle check point^52^, the regulation of export of unspliced mRNA forms^38^ and the establishment of perinuclear heterochromatin exclusion zones^30^. Tpr has an intriguing location that places this Nup as a bridge between the NPC and the underlying chromatin^29^,^30^,^53^. In this study we observed a strong decrease of infectivity (~1log) in Tpr-depleted cells (Fig. 2b,d), although HIV-1 nuclear translocation and integration were not affected (Fig. 2f,g). We characterized a specific role of Tpr in HIV-1 infection. HIV-1 but not MLV showed a defect in infectivity in stable clones depleted for Tpr (Fig. 3b). We also observed that Tpr depletion, which specifically reduces HIV-1 expression, did not affect global gene regulation (Fig. 3c–f; Fig. 4e; Supplementary Fig. 3). For this purpose, we analysed Jurkat cells that are stable cell lines derived from lymphocytes, are targets of HIV-1, and allow long testing conditions. Previous studies showed similar gene expression profiles for Jurkat cells and primary CD4+ T lymphocytes, allowing the use of Jurkat cells as model HIV target cells^54^. We observed that in Tpr-depleted Jurkat cells, unlike in control cells, HIV-1 integration exhibits weaker preference for DNase I HS sites and for the chromatin mark H3K36me3, which is associated with actively transcribed chromatin (Fig. 5a,c; Supplementary Fig. 4). Using super-resolution microscopy, we found that Tpr depletion results in a reduced density of H3K36me3 in the nuclear periphery beneath NPCs (Fig. 6). These results from two independent techniques support a model in which HIV-1 integration occurs preferentially in chromatin regions proximal to NPC and where Tpr remodels these regions in an active state favourable for HIV-1 replication. We further showed that the host cellular factor LEDGF/p75, which is important for HIV-1 integration in active chromatin, is stabilized at the nuclear periphery when Tpr is overexpressed and vice versa (Fig. 4). Recent studies showed that LEDGF/p75 directly interacts with H3K36me3^45^. We showed a reduction of this histone mark in HIV integration sites (Fig. 5) and near NPC (Fig. 6) when Tpr is depleted. Earlier studies reported that the absence of Tpr induces the presence of heterochromatin near the nuclear basket^30^. Since LEDGF/p75 is directly associated with H3K36me3 (ref. 45), Tpr depletion could provoke a displacement of LEDGF/p75, which in turn could explain the change in chromatin targets. Previous reports suggest that nuclear basket Nups cooperate with LEDGF/p75 for efficient viral replication^9^,^13^,^15^,^55^ (Supplementary Discussion). Our study suggests the existence of a complex formed near the nuclear basket, predominantly composed of Tpr, LEDGF/p75, and active chromatin regions. LEDGF/p75 is randomly distributed in the nucleus^56^ bound to H3K36me3 (ref. 45). According to our data (Figs 4, 5, 6), it is possible that a major recruitment of H3K36me3 near NPCs occurs when Tpr is overexpressed, which could lead to the observed increase of LEDGF/p75 underneath NPCs as shown in Fig. 4. Whether the interactions among these factors are direct or not remains an appealing question for future investigations.

We speculate that the absence of Tpr reproduces a phenotype similar to that of persistent viral infected cells, in which the virus integrates but does not replicate. A possible hypothesis about HIV-1 persistence is that proviral transcription is influenced by chromatin structure with a potential involvement of histone remodelling at the viral integration site. This study could, thus, have implications for the understanding of cellular reservoirs, populations of persistent viral infected cells, which remain one of the most important obstacles to curing HIV-1. In summary, this work adds an important piece to our understanding of HIV-1 replication mechanisms and will be relevant for future studies of the impact of chromatin topology on other host–pathogen interactions.

## Methods

### Cells, lentiviral vector carrying shRNA and HIV-1 infection

The jurkat cells are CD4+ human T cells. The 293T cells are human embryonic kidney cells. The HeLa P4-CCR5 reporter cells are HeLa CD4+ CXCR4+ CCR5+ carrying the LacZ gene under the control of the HIV-1 LTR promoter^32^. Complementary oligonucleotide coding for shRNA cassette (5′- GGTGGAGAGCGAACAACAG -3′)^33^ against Tpr or against Nup153^13^ were first annealed and cloned into BglII/HindIII of pSUPER (OligoEngine) downstream of the H1 promoter. The H1-shRNA cassettes were then inserted in the 3′ U3 region of the HIV-1-derived vectors (LV-shRNA), TRIP-GFP or TRIPsym vectors, which are ΔU3. These LVs contain the *cis*-acting sequences required for formation of the central DNA Flap. The TRIPsym has the DNA Flap exactly located on the centre of the transfer vector, to increase the efficiency of transduction. LV-shRNAs carrying GFP were titered in HeLa P4-CCR5 cells using flow cytometry to assess GFP expression at 3 days post-transduction (p.t). In addition, TRIP-GFP and TRIPsym with shRNA (LVshRNA) were tittered by p24 ELISA according to the manufacturer’s instructions (Perkin Elmer).

HeLa P4-CCR5 and Jurkat cells were transduced with LV-shRNA against Tpr or Nup153 at different MOI up to 100 to generate knockdown cells (Figs 1a,b and 2a,c; Supplementary Fig. 7). For reasons of viability, half-life and stability of each nucleoporin, Tpr and Nup153 KD cells were used at 2–3 days p.t. Stable Tpr depleted and control HeLa P4CCR5 were obtained by limiting dilution (Fig. 3a; Supplementary Fig. 7). Lentiviral vectors were produced by transient transfection of 293T cells using calcium phosphate coprecipitation with NL4.3 Luc ENV^−^ or NL4.3 RFP ENV^−^ (luciferase or RFP genes in place of Nef) and cotransfection with the VSV-G envelope expression plasmid pHCMV-G (VSV-G). The viruses collcted from 293T cells 48 h post transfection were treated with 25 U ml^−1^ of DnaseI (Roche) and with 100 mM MgCl2 at 37 °C for 30 min. Virus normalizations were performed by p24 ELISA according to the manufacturer’s instructions (Perkin Elmer). Retroviral vector, MLV-Luc, derived from Moloney was produced by cotransfection with calcium phosphate of pFBluc 10 μg, pCG gag-pol 10 μg, pMD2 VSV-G 2 μg.

### Luciferase assays

Luciferase (Promega) activity was measured 48 h p.i. according to manufacturer’s instructions, using a microplate fluorimeter (Victor, Perkin Elmer). Protein quantification by Bio-Rad protein assay was carried out on the same lysates to normalize the luciferase data for protein content.

### Quantitative PCR

Viruses were treated for 30 min at 37 °C with 1,000 U of DnaseI (Roche). Five micromolar of nevirapine used in infected cells as control of the experiment. Total cellular DNA was then isolated using the QIAamp DNA micro kit (QIAGEN) at 7 and 24 h p.i.. Late reverse transcription products at 7 h p.i. were measured by real-time PCR using Sybergreen and primers against luciferase (5′- GAATCCATCTTGCTCCAACAC -3′; 5′- TTCGTCCACAAACACAACTC -3′), which is exclusively in the HIV-1-Luc and not in the LVshRNA previously used to generate Tpr KD or control cells. 2LTR containing circles were detected using primers MH535/536 and probe MH603 (ref. 34), using as standard curve the pUC2LTR plasmid, which contains the HIV-1 2LTR junction. Integration was assessed by Alu-PCR, using primers designed in the U3 region of LTR^13^,^14^,^57^, which is deleted in the LVs carrying shRNA but not in the LTR of HIV-1 used to challenge Tpr depleted and control cells.

### Binding of Nups to *in vitro* HIV-1 CA–NC complexes

The 293 T cells were transfected with plasmids expressing TPR-GFP or Nup153-GFP proteins. Forty-eight hours after transfection, cell lysates were prepared as follows: previously washed cells were resuspended in hypotonic lysis buffer (10 mM Tris, pH 7.4, 1.5 mM MgCl_2_, 10 mM KCl and 0.5 mM DTT). The cell suspension was frozen and thawed, and incubated on ice for 10 min. Afterwards, the lysate was centrifuged at maximum speed in a refrigerated Eppendorf micro centrifuge (~14,000 *g*) for 5 min. The supernatant was supplemented with 1/10 volume of 10 × PBS and then used in the binding assay. To test binding, 5 μl of CA–NC particles assembled *in vitro* were incubated with 200 μl of cell lysate at room temperature for 1 h. A fraction of this mixture was stored (input). The mixture was spun through a 70% sucrose cushion (70% sucrose, 1 × PBS and 0.5 mM DTT) at 100,000 *g* in an SW55 rotor (Beckman) for 1 h at 4 °C. After centrifugation, the supernatant was carefully removed and the pellet resuspended in 1 × SDS–PAGE loading buffer (pellet). The level of TPR-GFP and Nup153-GFP proteins were determined by western blotting with anti-GFP antibody (Clontech #632592 1:1000). The level of HIV-1 CA–NC protein in the pellet was assessed by western blotting with anti-p24 CA antibody (Abcam ab9071, 1:2,000; Fig. 1f, Supplementary Fig. 7) and a secondary Ab anti-mouse IRDye-680-it926–68020 LICOR (1:1,000). Blots were analysed using LICOR technology. The HIV-1 CA–NC protein was expressed, purified and assembled *in vitro* by diluting the CA–NC protein to a concentration of 0.3 mM in 50 mM Tris–HCl (pH 8.0), 0.5 M NaCl and 2 mg ml^−1^ DNA oligo-(TG)50. The mixture was incubated at 4 °C overnight and centrifuged at 8,600 *g* for 5 min. The pellet was resuspended in assembly buffer (50 mM Tris–HCl (pH 8.0), 0.5 M NaCl) at a final protein concentration of 0.15 mM^13^,^37^,^58^, and stored at 4 °C.

### Transcriptomic analysis

RNA sequencing has been performed to total RNA isolated from the following samples: Clone 2, Clone 6, Tpr KD HeLa and control (LV-shRNA MOI 50) and Tpr KD Jurkat and control (LV-shRNA MOI 50), using the RNeasy mini kit Qiagen. Total RNAs were qualitatively checked using the Agilent TapeStation and the RNA ScreenTape (Agilent Technologies, Santa Clara, CA, USA) and quantitated using the NanoDrop 1000 spectrophotometer (Thermo Scientific, Wilmington, DE, USA). For each sample, 1,000 ng of total RNA was prepared to obtain an Illumina indexed library, following the protocol TruSeq RNA sample Preparation V2. Indexed libraries were then sequenced using the Illumina platform MiSeq and the V3 sequencing chemistry. Data were analysed as following. For each sample, the reads were mapped on the annotated human genome reference sequence (Hg 19 release) and the Reads Per Kilobase of transcript per Million mapped reads (RPKM) values were calculated using the CLC Workbench software (CLC Bio-Qiagen, Aarhus, Denmark). For a visual comparison of the transcriptome profiles, a scatter plot were designed reporting RPKM values observed in all ~36,000 genes annotated. To avoid problem due to RPKM equal to zeros and to ensure a better visualization, a log_2_ (RPKM+1) transformation was applied (Fig. 3d–f). To assess the similarity between the Tpr KD and control samples, Pearson correlation coefficient was calculated. Graphs and indexes calculations were performed using the R software (version 3.0.1).

### RT–PCR

Total RNA was extracted from clones 2 and 6, Tpr KD and control HeLa cells, Tpr KD and control Jurkat cells. RT–PCR has been performed on these samples using the SuperScript III Platinum SYBR Green One-Step qRT–PCR Kit. Primers used were the following: PSIP1 (5′- GTTACTTCAACCTCCGATTCTG -3′; 5′- TTGATGTTTCTCGCTTCTTCTC -3′), TPR (5′- CTT GTAAATTGGCTCTGAATGG -3′; 5′- AGACTTGTGAATGAAACC CGA -3′), GAPDH (5′- CAT TTC CTG GTA TGA CAA CGA -3′, 5′- CTT CCT CTT GTG CTC TTG CT -3′), ACTIN (5′- AAG ATC AAG ATC ATT GCT CCT CC -3′, 5′- GTC ATA GTC CGC CTA GAA GCA -3′), TRIM24 (5′- AGC AAA CGA CTG ATT ACA TAC C -3′, 5′- TTT GAG CCC AGA AAC TAG GA -3′), SNX27 (5′- CAA GTA TAT CAG GCT ATC GCA -3′, 5′- TCT GAA TGT AGA GTT TGT GAG G -3′).

### Sample preparation for microscopy

Cells were seeded onto 12 mm diameter coverslips in 24-well plates the day before fixation or infection. Cells were fixed in 2% paraformaldehyde for 10 min, treated with 50 nM NH4Cl for 10 min, permeabilized with 0.5% triton for 30 min and blocked with 0.3% bovine serum albumin (BSA). All incubations were carried out at room temperature and were followed by five PBS washes. Cells were incubated with primary antibodies for 1 h and secondary antibodies for 30 min. Antibodies were diluted in 0.3% BSA. Nuclei were stained with Hoechst (Invitrogen). Finally, cells were mounted onto glass slides (Thermo Scientific) with Prolong Antifade (Life Technologies).

### Western blotting and confocal microscopy

The deletion mutant GFP-Nup153ΔFG generated by DNA restriction with XbaI of the GFP-Nup153, GFP-Nup153, pGFP-Nup98, pHA-Np153 (Euroscarf), pGFP-Tpr (Addgene), pcDNA IN-HA (kind gift of S. Emiliani), pHA-LEDGF/p75, pCEP75Flag (kind gift of P. Cherepanov), pAcGFP1-IN and pC2GFP (Clontech), pCMV Tat and pAcGFP1-IN (kind gift of X. Yao) plasmids were transfected using lipofectamine 2000 in HeLa cells or using calcium phosphate in 293T cells. Proteins were extracted on ice from wild type and KD cells using RIPA buffer (20 mM HEPES pH 7.6, 150 mM NaCl, 1% sodium deoxycholate, 1% Nonidet P-40, 0.1% SDS, 2 mM EDTA, complete protease inhibitor (Roche Diagnostics)), and protein concentration was quantified using the Dc Protein Assay (Bio-Rad Laboratories) with BSA as standard. Hundred micrograms of total protein lysate was loaded onto SDS–PAGE 6% Tris-glycine or 4–12% Bis Tris gels (Invitrogen). Revelation was carried out using the ECL Plus western blotting kit (GE Healthcare). Primary antibodies used for western blotting (WB) were anti-Tpr (Santa Cruz sc-101294 WB 1:1,000, IF 1:100), anti- Nup153 (SA1, kind gift from B. Burke, WB 1:500, IF 1:10), anti-GFP (Clontech #632592 1:1000), anti-HA (Covance MMS-101 R 1:1000), anti-Flag HRP conjugated (Sigma A8592 1:1000), anti-lamin A/C (Santa Cruz sc-7292 1:500), anti- LEDGF/p75 (Bethyl A300–847A 1:200). Secondary conjugated antibodies used for western blotting were Beta Actin HRP conjugated antibody (Abcam, #8226 1:2,500), anti-mouse IgG HRP (GE Healthcare NA931 1:5,000) and anti-rabbit IgG HRP (GE Healthcare, NA 934 1:5,000). Secondary conjugated antibodies for IF were anti-mouse Cy3 (GE Healthcare PA43002 1:300). Hoechst (Invitrogen 1:10,000) was used to stain nuclei. Western blots were quantified using ImageJ or MYImageAnalysis (Thermo Scientific). Confocal microscopy was carried out using a Zeiss LSM700 confocal microscope with a 63 × objective, using identical laser and exposure times for all samples, including negative controls (only secondary antibodies). Four independent experiments were performed.

### HIV-1 integration sites distribution

Twenty million Jurkat cells (control and Tpr KD) were infected with 10 μg of p24 antigen of NL4.3-Luc ENV^−^. Three days later, genomic DNA was extracted by QIAamp DNA micro kit (QIAGEN) and digested with the four-cutter enzymes *Bfa*I and *Bgl*II to prevent the amplification of internal 3′ LTR fragments, using the protocol described in ref. 13. Raw sequence reads were processed through an automated bioinformatic pipeline that eliminated small and redundant sequences, and were mapped to the UCSC hg19 release of the human genome^59^. Sequences with 90% or greater identity to the human genome were considered genuine integration sites. To investigate the relationship between integration sites (ISs), DNaseI hypersensitive sites and histone methylation profiles, we used data retrieved from NGS on Jurkat cells and CD4^+^ T cells available from http://www.uwencode.org/data/releases (GSM736501,GSM945267 (ref. 39), http://dir.nhlbi.nih.gov/papers/lmi/epigenomes/hgtcell.aspx^60^, which were properly remapped and adapted from the hg18 to the hg19 version of the human genome using the LIFTOVER tool available on the UCSC genome browser. From the raw signal bed files, we calculated and analysed the distances of each feature tag from every integration site considering a ±50 kb window centred on ISs. This task was performed on three data sets: control, Tpr KD and a random data set composed of 10,000 integrations *in silico* generated according our experimental setting. To visualize associations between ISs and aforementioned genomic features at short and wide ranges, we performed two different analysis. To highlight wide-range behaviour, the distribution of all distances (within the −50 kb window) was plotted as a histogram with bins of 100 bp. In addition a Gaussian kernel density estimator with Sheather and Jones bandwidth selection was calculated and superimposed to the histograms (Fig. 5c,d; Supplementary Fig. 4b). To test for statistical differences of the distance distributions between control cells, Tpr KD and random ISs, we performed three Kolmogorov–Smirnov tests for each genomic feature: (control versus Tpr KD, control versus random, Tpr KD versus random). We analysed whether two empirical distributions of distances could be assumed to be drawn from the same underlying distribution that was for all <2.2e−16. To compare differences in short-range association among data sets, we considered only annotations taking distances in absolute values <2,000 b.p.s. and bin size equal to 200 b.p.s. (Fig. 5a,b; Supplementary Fig. 4a). To compare eventual differences in observed proportions, we added a confidence interval. In addition, a set of Pearson's *χ*^2^-test was performed (*P*<2.2e−16). To investigate the distribution of ISs with respect to genes, we considered known genes according to the UCSC definition and annotated as TSS-proximal if it was within±2.5 kb from a TSS, intragenic when inside a gene at>2.5 kb from the TSS, and intergenic in all other cases. Results are summarized in Supplementary Table 1.

### Dual-colour STORM imaging: H3K36me3 and NPCs

To analyse the localization of histone modifications relative to nuclear pores (Fig. 6), we performed dual-colour stochastic optical reconstruction microscopy (STORM)^40^ using a homemade STORM system^61^,^62^,^63^. Dual-colour labelling was achieved using primary antibodies, anti-Nup153 (SA1, kind gift from B. Burke; 1:10) and anti-H3K36me3 (Cell Signaling #9763 1:200), secondary antibodies coupled to the synthetic dyes Cy5 Jackson #711-605-152 (1:300) and Alexa568 Life Technologies A11031 (1:300). To achieve robust fluorophore blinking (a requirement for STORM), we employed a commonly used oxygen scavenger buffer^64^.

Coverglasses were covered with a parafilm sheet, in which we cut a small square hole, and filled it with the oxygen scavenger. Four colours Tetraspec beads (100 nm diameter) were added to the sample as fiducial markers to estimate sample drift and chromatic aberrations during imaging. The coverslip was deposited on top of the buffer-containing parafilm hole and hermetically sealed using nail polish.

Cells were selected for STORM imaging based on wide field images of the nucleoporin signal (Alexa568 channel). The focal plane was chosen such as to visualize an annulus of NPC corresponding to an equatorial cut through the nucleus. First, using laser illumination at 642 nm and a filter matched to Cy5, we acquired 20,000–30,000 raw images of blinking fluorophores. We then acquired a similar number of images with a filter matched to Alexa568 using laser excitation at 561 nm.

After acquisition of raw images, fluorophore positions were computed using PALMTT, a modified version of the Matlab-based 2D Gaussian fitting algorithm MTT^65^. We used custom-made Matlab algorithms to estimate and correct sample drift based on the estimated trajectories of fluorescent beads. Similarly, we used the bead fluorescence in the two colour channels to estimate and correct for lateral chromatic shifts using an affine transformation.

We verified our reconstruction tools on dual-colour STORM images of Nup153-A568 and Nup214-Cy5 (Supplementary Fig. 5).

The high resolution of the STORM images allowed us to clearly identify individual NPCs (Fig. 6a,b). We used custom-made Matlab tools to manually outline the NE (by drawing a polygon through the nucleoporin signal) and click on the locations of individual NPCs as well as positions on the NE located between neighbouring NPCs. The manual selection of these locations (and outlining of the NE) was done only on the nucleoporin-Cy5 image (without visualization of the histone-A568 signal), thus preventing potential user biases. Our tool, then, automatically defined 50 nm wide and 1 μm long rectangles passing through these points and perpendicular to the local NE outline (Fig. 6b). To assess the localization of histone modification H3K36me3 relative to the NPCs, we, then, computed the coordinate of each detected A568 position projected along the long axis of the rectangle. For rectangles passing through NPCs, the coordinate zero was defined as the position of the NPC, which we refined by fitting a gaussian to the histogram of position coordinates computed from the nucleoporin-Cy5 signal. To assess the statistical distribution of histone marks across the NE, we further proceeded as follows: for each rectangle, we counted the A568 positions in 5 nm stripes perpendicular to the rectangle length. We, then, binarized the data, such that each 5 nm bin was given a value of 0 if it contained no A568 localization and 1 otherwise. This was done to reduce the effect of large variations in the number of blinking events between different molecules. We, then, smoothed the resulting binary profile by averaging over 10 data points, resulting in 50 nm bins. Finally, for each 50 nm bin, we computed the median of the filtered profile over all rectangles (separately for those passing through NPCs and those passing between NPCs). These median profiles are reported in Fig. 6c.

## Author contributions

F.D.N. conceived and designed the study. M.L., C.Z. and F.D.N. conceived and designed the experiments of super-resolution microscopy, which were carried out by M.L. and F.D.N., M.L. and C.Z. developed tools for image analysis. F.D.N., P.S. and P.C. performed the virology and molecular biology experiments. N.C. and F.D.N. conceived and designed confocal microscopy experiments. T.F. and F.D.G. performed CA–NC binding assay. E.R., M.S. performed the 454 pyrosequencing, RNA sequencing and sequencing data analysis. D.P. and C.D.S. performed bioinformatics analysis. F.D.N. and C.Z. wrote the manuscript.

## Additional information

**How to cite this article:** Lelek, M. *et al*. Chromatin organization at the nuclear pore favours HIV replication. *Nat. Commun.* 6:6483 doi: 10.1038/ncomms7483 (2015).

## Supplementary Material

Supplementary Information Supplementary Figures 1-7, Supplementary Table 1, Supplementary Discussion Supplementary Methods and Supplementary References
