# Supplementary material for: Chromatin organization at the nuclear pore favours HIV replication
Source: Nat Commun. 2015 Mar 6;6:6483. doi: 10.1038/ncomms7483 (PMC4366494; doi:10.1038/ncomms7483)
Supplement: Supplementary Information — Supplementary Figures 1-7, Supplementary Table 1, Supplementary Discussion Supplementary Methods and Supplementary References [file ncomms7483-s1.pdf]

a

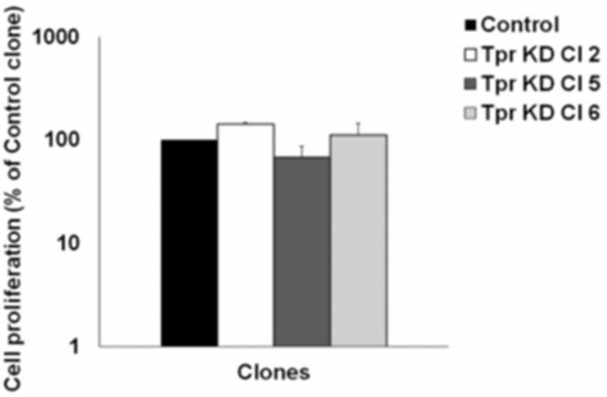

b

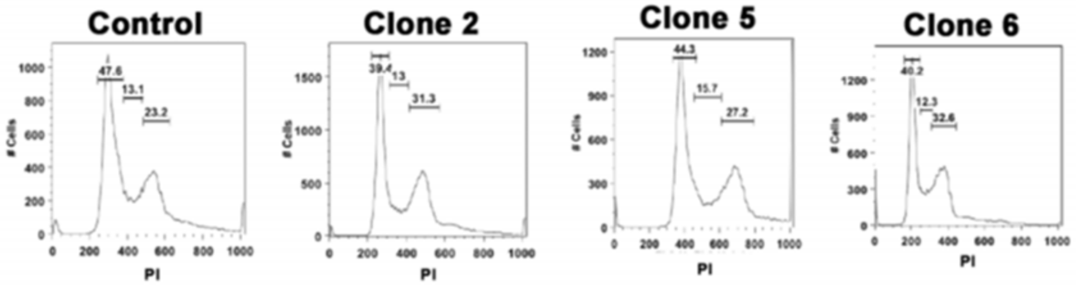

Supplementary Figure 1. Clone proliferation and cell cycle. a) Colorimetric method CellTiter 96® AQueous One Solution Cell Proliferation Assay has been used for determining the number of viable clones in proliferation. **b)** Clone cycling was assessed by propidium iodide labelling analysed by flow cytometry (propidium iodide in x axes and cell counts in y axes).

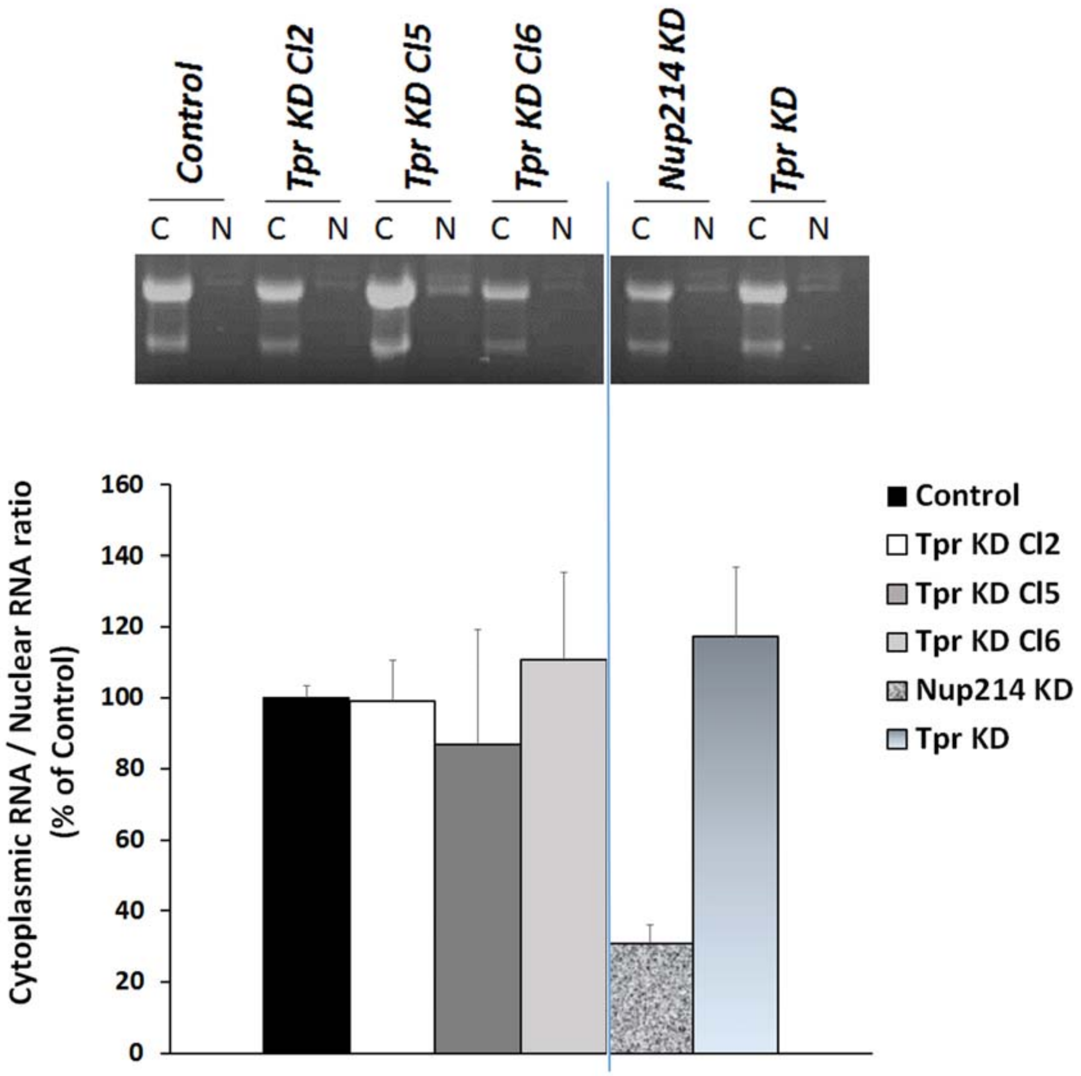

Supplementary Figure 2. Ratio between RNA cytoplasmic and nuclear fractions. Cytoplasmic and nuclear RNA were quantified with a Nanodrop ND-1000, loaded on 1% agarose-formaldehyde gel and quantified by ImageJ as shown in the histogram of the mean ratio of cytoplasmic/nuclear RNA +/− SD. Stable clones, Nup214 HeLa bulk and Tpr KD HeLa P4CCR5 bulk are respectively shown.

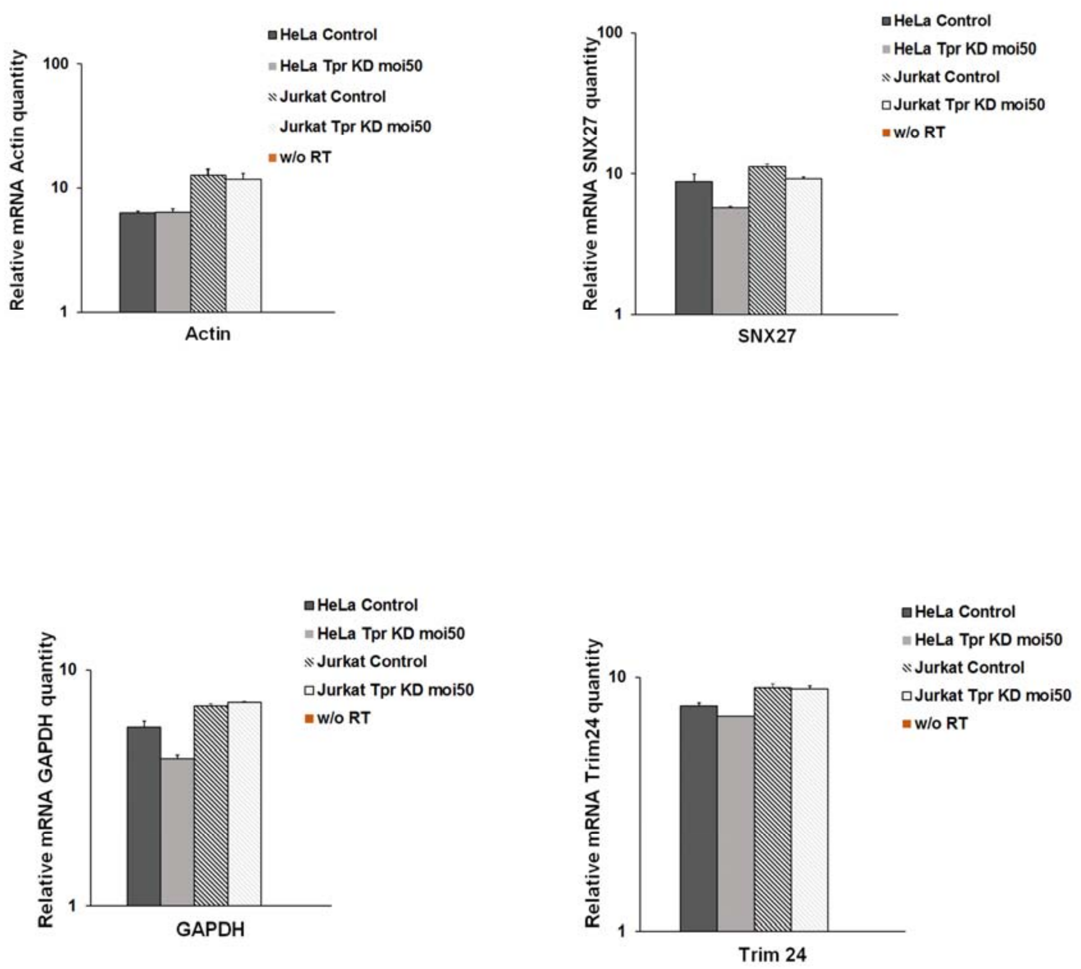

Supplementary Figure 3. Profile of expression of specific genes. RT PCR, relative mRNA quantity of Actin, GAPDH, Trim24 and SNX27 in Tpr depleted vs control HeLa and Jurkat cells. RT PCR without RT enzyme has been used as control in all reactions. Standard deviations are shown of duplicate experiments.

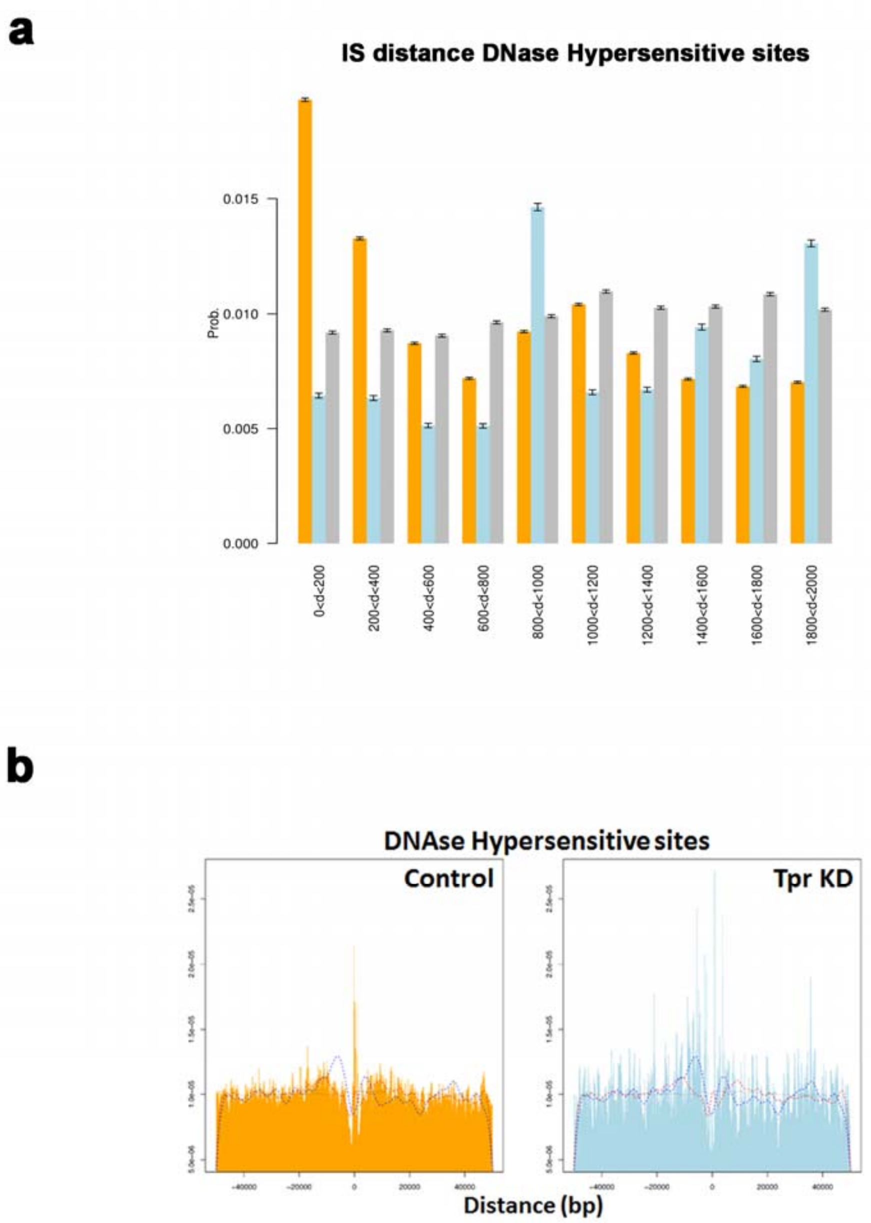

Supplementary Figure 4. HIV-1 integration sites and genomic feature. a) Histograms show the distribution of absolute genomic distances to DNaseI hypersensitive of HIV-1 integration sites, with bin size of 200. **b)** Frequency of ISs from DNaseI hypersensitive as in **figure 5c,d**.

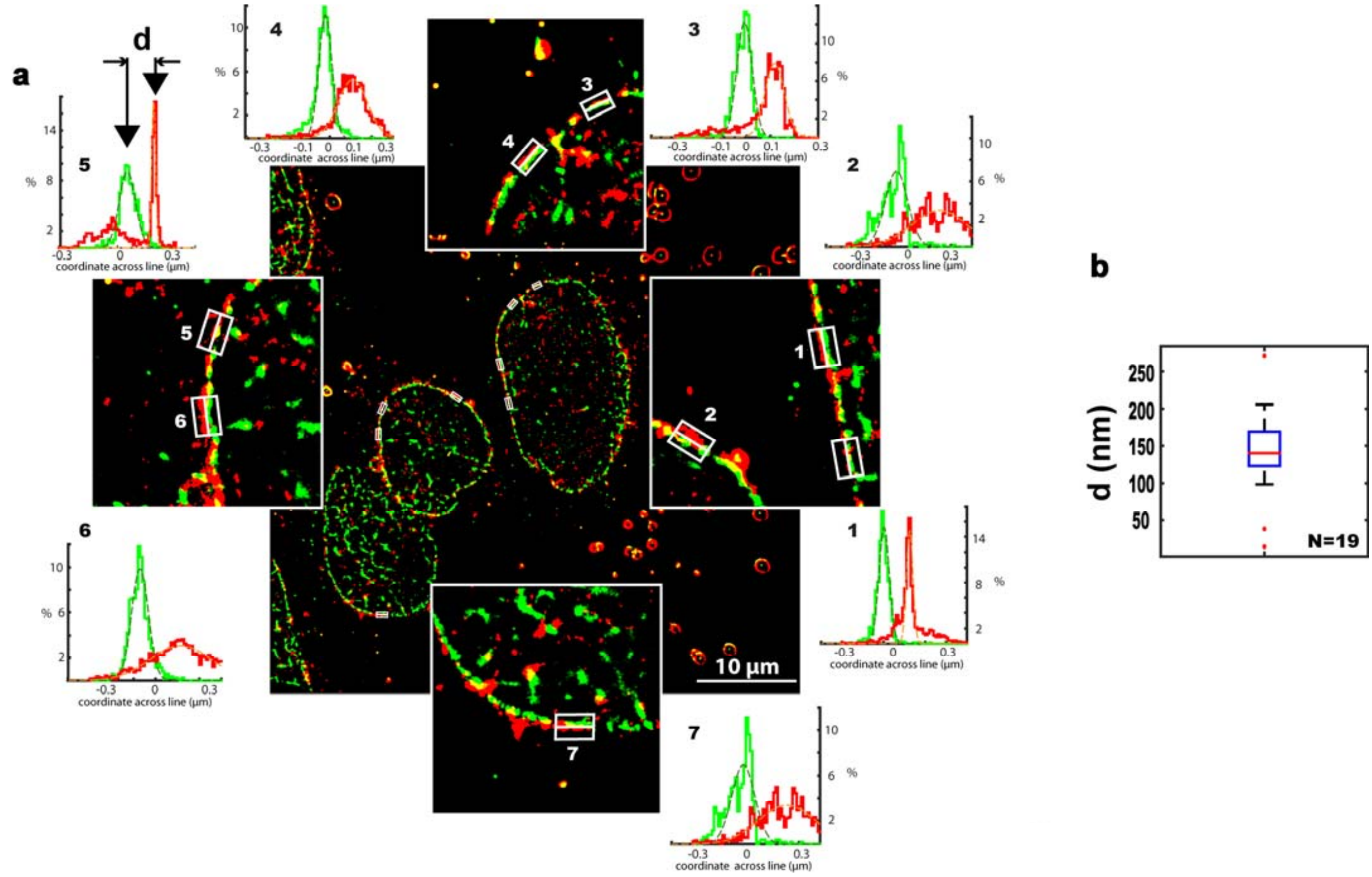

Supplementary Figure 5. Dual colors STORM of two nucleoporins. To verify the imaging procedure, we simultaneously imaged Nup153 labelled with A568 (green) and Nup214 labelled with Cy5 (red). Multicolor fluorescent beads were used to computationally correct sample drift and chromatic shifts. The two colors channels show a degree of overlap, as expected. **a**) The distance (d) between Nup214 and Nup153 has been processed from scatter plots image. Boxes (n=19) were manually selected in opposite sites along the nuclear membrane. Zooms (8 µmx8µm) around the main image and the corresponding boxes are represented. For each box, histograms and their gaussian fit are plotted for each coloured histograms. The distance is calculated from maximum of the gaussian fits. **b**) Boxplot of the distance Nup214-Nup153.

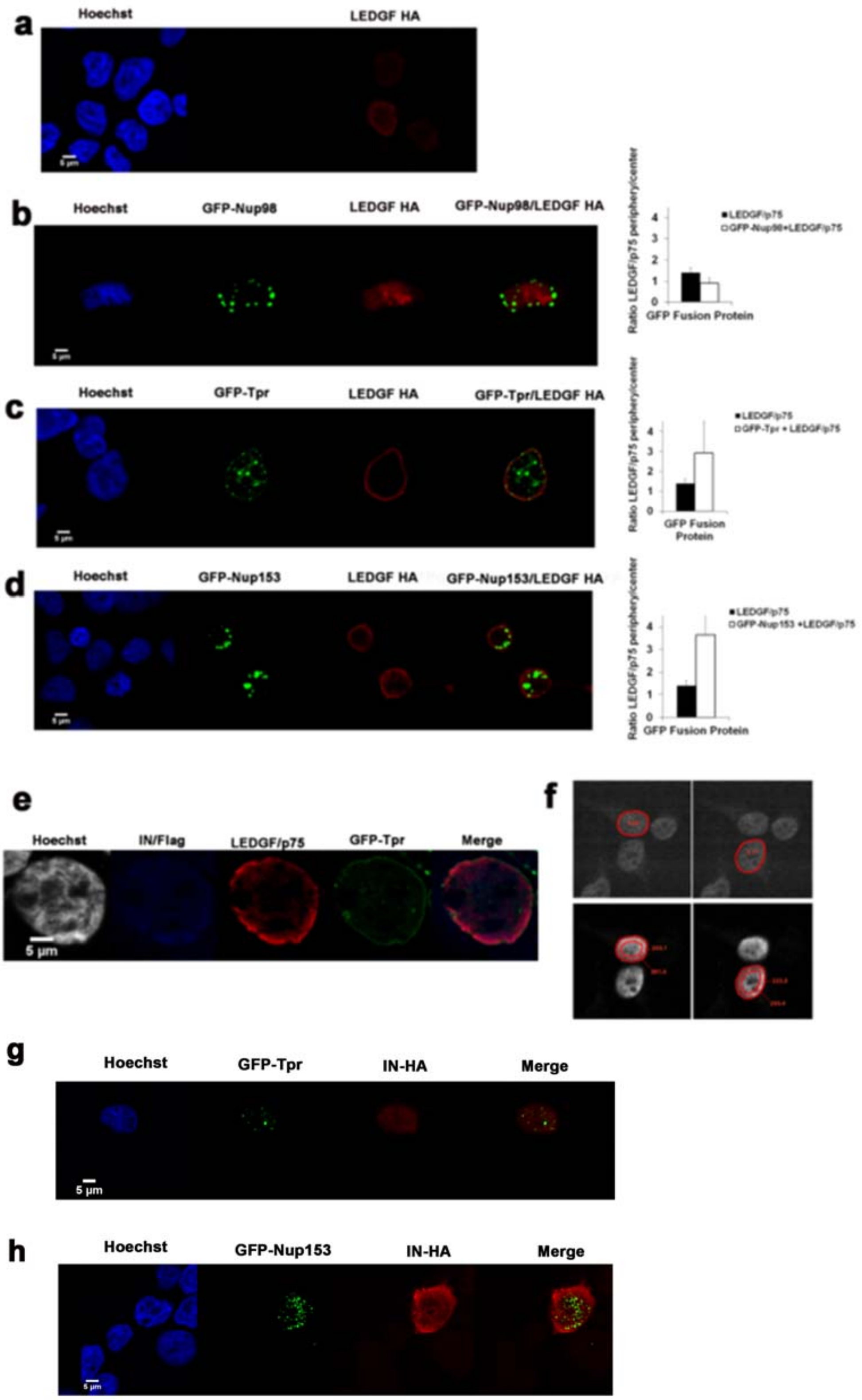

a
Hoechst
LEDGF HA
b
Hoechst
GFP-Nup98
LEDGF HA
GFP-Nup98/LEDGF HA
Ratio LEDGF/p75 periphery/center
LEDGF/p75
GFP-Nup98+LEDGF/p75
GFP Fusion Protein
c
Hoechst
GFP-Tpr
LEDGF HA
GFP-Tpr/LEDGF HA
Ratio LEDGF/p75 periphery/center
LEDGF/p75
GFP-Tpr + LEDGF/p75
GFP Fusion Protein
d
Hoechst
GFP-Nup153
LEDGF HA
GFP-Nup153/LEDGF HA
Ratio LEDGF/p75 periphery/center
LEDGF/p75
GFP-Nup153 +LEDGF/p75
GFP Fusion Protein
e
Hoechst
IN/Flag
LEDGF/p75
GFP-Tpr
Merge
5 µm
f
g
Hoechst
GFP-Tpr
IN-HA
Merge
5 µm
h
Hoechst
GFP-Nup153
IN-HA
Merge

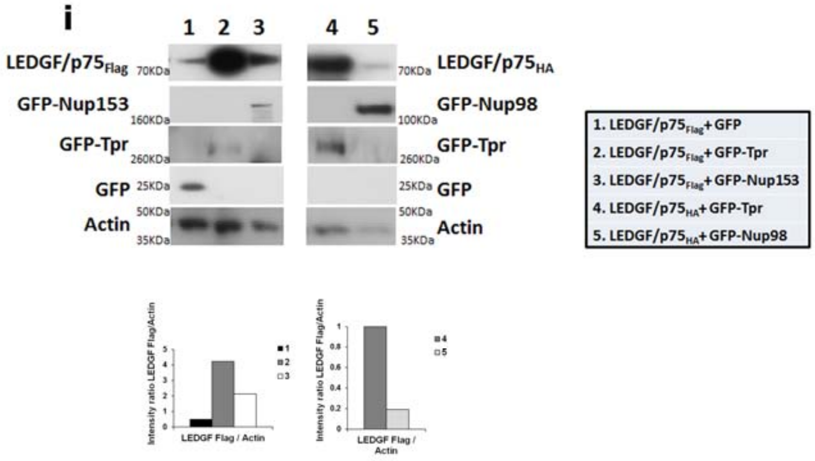

Supplementary Figure 6. Nuclear localization of LEDGF/p75 in cells that overexpress GFP-Nup98, GFP-Tpr, GFP-Nup153. 293T cells were transfected with **a)** LEDGF/p75-HA alone and **b)** co-transfected with LEDGF-HA and GFP-Nup98 **c)** co-transfected with LEDGF-HA and GFP-Tpr **(d)** co-transfected with LEDGF-HA and GFP-Nup153, **e)** co-transfected with LEDGF-HA, GFP-Nup153 and IN-Flag. As control **g)** GFP-Tpr and **h)** GFP-Nup153 were co-transfected with a plasmid coding for another protein fused to HA tag, the integrase of HIV-1 (IN-HA). LEDGF-HA and IN-HA were labelled with a primary antibody against HA and a secondary antibody conjugated with Cy3. In panel e LEDGF-HA was labelled with an antibody against LEDGF (Bethyl) and the IN-Flag with an antibody anti-Flag (Sigma), secondary antibody were conjugated with Cy3 and Cy5. **f)** We quantified the intensity in the nuclear periphery and the nuclear center in the 2 channels (green for the GFP fused proteins and red for LEDGF/p75) in~20 cells per condition (see Materials & Methods). The nuclear periphery is defined manually by drawing a polygonal shape based on the Hoechst staining (blue channel). The mean GFP intensity of GFP (green channel) computed inside this shape. The nuclear periphery is defined as a 2 μm wide band from the polygonal outline. The mean intensity of LEDGF/Cy3 (red channel) is computed separately in the peripheral and central region. The bar charts in **(b,c,d)** show the average ratio of LEDGF/p75 intensity of the nuclear periphery to the nuclear center, with respective standard deviations. **i)** Enhancement of LEDGF/p75 by overexpression of GFP-Tpr. 293T cells were co-transfected respectively with LEDGF/p75-Flag and GFP **(lane 1)**, LEDGF/p75-Flag and GFP-Tpr **(lane 2)**, LEDGF/p75-Flag and GFP-Nup153 **(lane 3)**, LEDGF/p75-HA and GFP-Tpr, as internal control for the second gel **(lane 4)**, LEDGF/p75-HA and GFP-Nup98 **(lane 5)**. All lanes were normalized by actin. Bar charts show the intensity ratio of LEDGF/p75 to actin quantified using MyImageAnalysis software (ThermoFisher).

Fig 1a

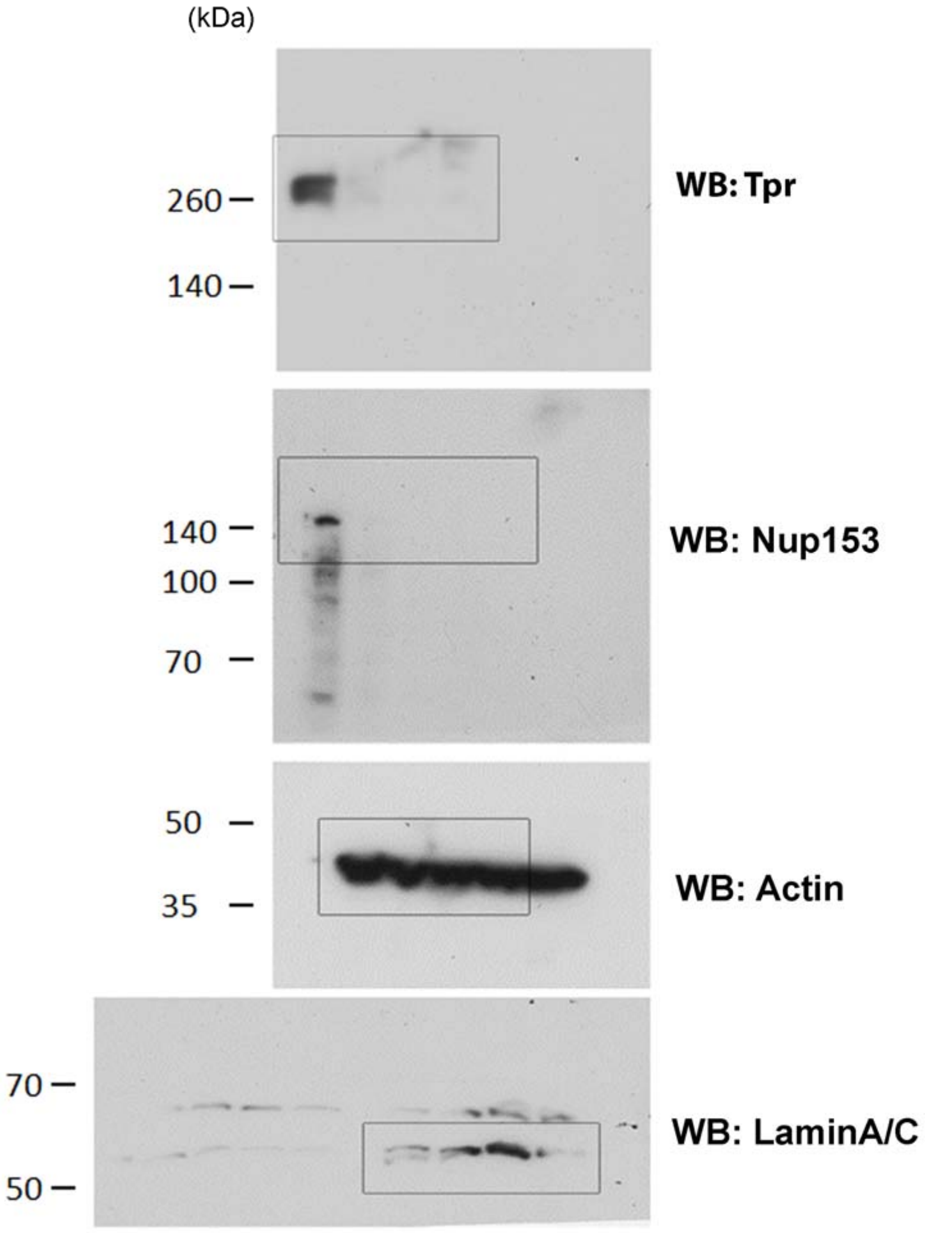

Supplementary Figure 7. Gel scans of most important western blots with indicated number of figure and areas of selection.

Fig 1c

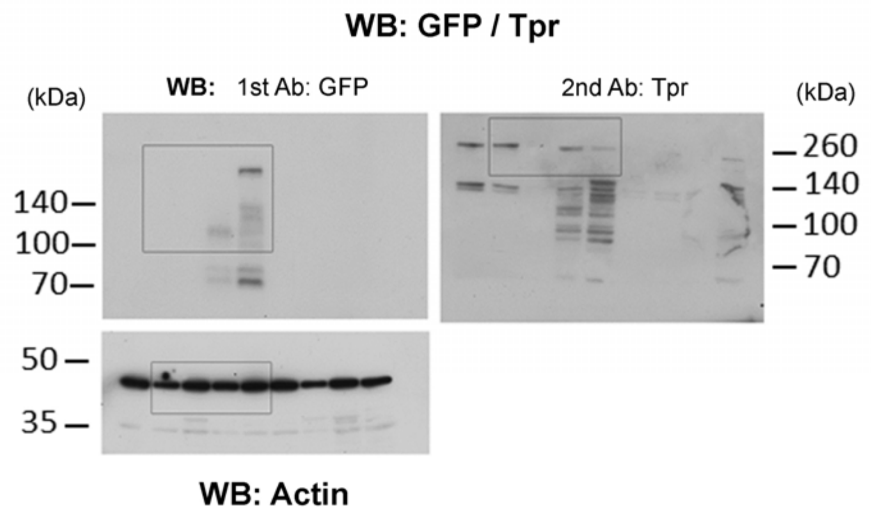

Fig 1f

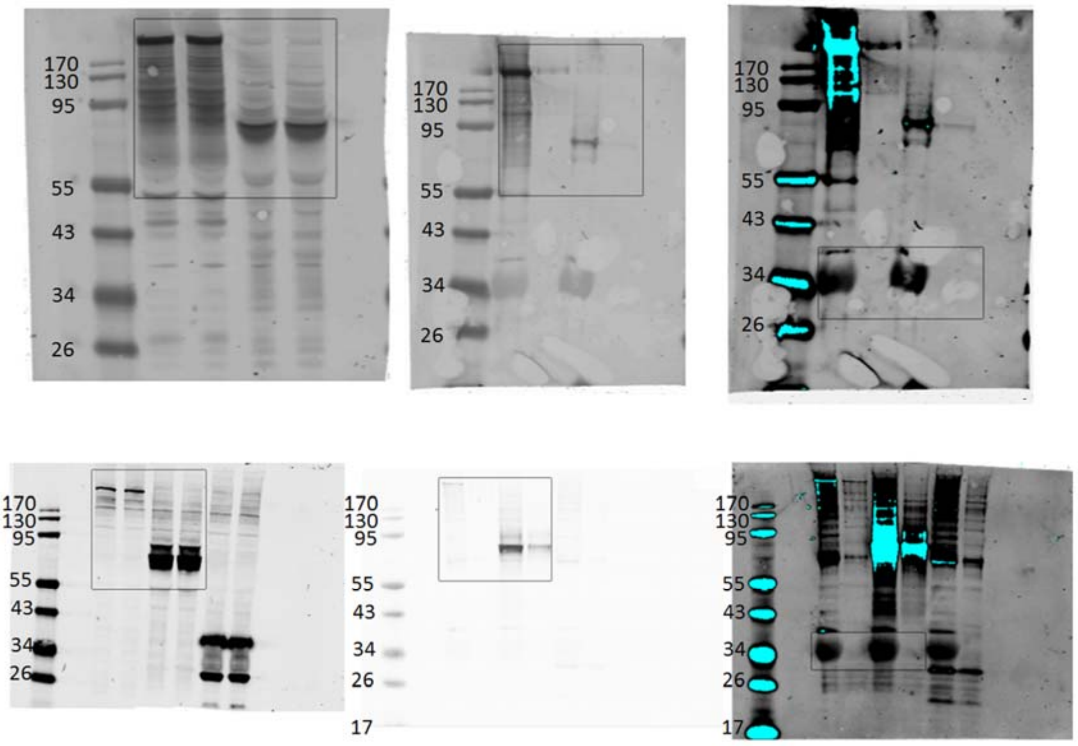

Supplementary Figure 7 continued

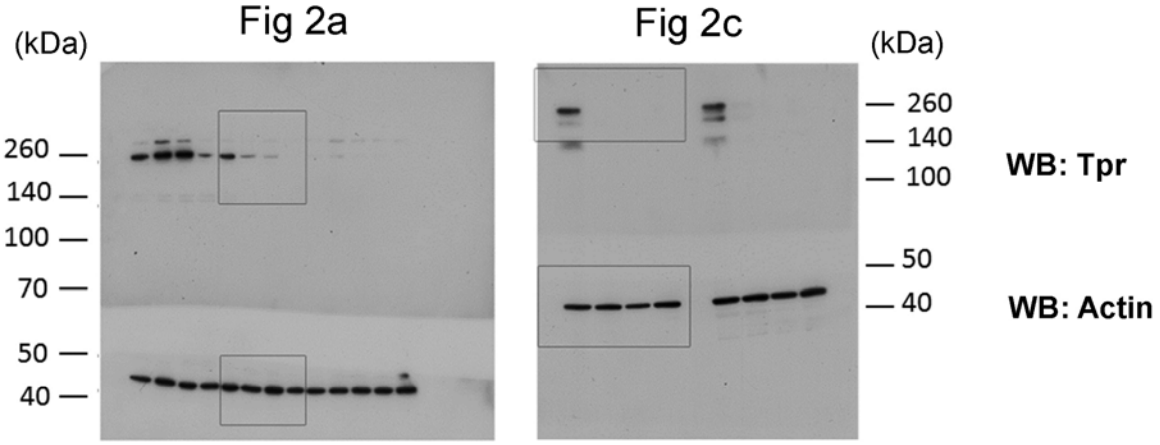

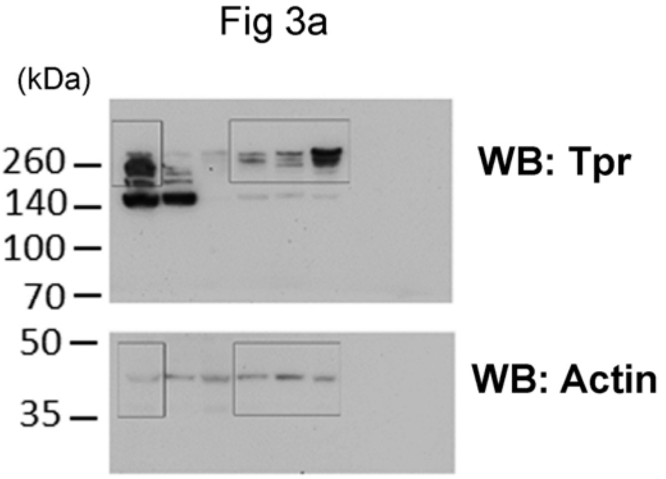

Supplementary Figure 7 continued

Supplementary Table 1. HIV-1 integration sites distribution

Chromatin regions	Jurkat Control	Jurkat Tpr KD	Random
% Intragenic integrations (±50Kb)	76.8	72.9	38.0
% Intergenic integrations (±50Kb)	19.9	23.5	59.0
% TSS-proximal integrations (±50Kb)	3.3	3.6	3.0

Supplementary Discussion

HIV-NPC-chromatin complex.

The first evidence for the role of LEDGF/p75 in HIV-1 target site selection comes from LEDGF/p75 knockdown (KD) cells that showed reduced frequencies of HIV-1 integration into active genes[1]. Our Tpr KD system shares some similarity with LEDGF/p75 KD cells, especially in driving proviral integrations in unusual chromatin sites (**Fig.5**). Previous studies suggested that LEDGF/p75 helps the virus to integrate in the body of actively transcribed genes, while nuclear basket Nups are involved in directing HIV-1 to accessible chromatin sites underneath the NPC[2,3,3,4]. We determined integration sites by high throughput pyrosequencing and found less integrations in intragenic and more in intergenic regions, in Tpr depleted Jurkat cells with respect to control cells (**Supplementary Table1**). Additionally, we observed a reduction of H3K36me3 near integration sites in Tpr depleted cells with respect to control cells, while H3K4me3 was excluded in both cases (**Fig.5**). The histone post-translational modification, H3K36me3, is highly represented near HIV-1 integration sites, while H3K4me3, an epigenetic mark of regulatory regions (enhancers and promoters), is under-represented at integration sites in HIV-1 infected cells[5]. We also know from previous reports that LEDGF/p75 directly binds H3K36me3 through the PWWP domain[6,7]. Furthermore, chimeric constructs of LEDGF/p75, in which the PWWP domain is replaced by domains interacting with heterochromatin, like CBX1, are able to address the viral genome towards heterochromatin sites[8]. This suggests the ability of HIV-1 to integrate in less accessible chromatin regions. Other studies are in agreement with the possibility to retarget HIV integration away from active genes[8,9,10,11]. Experiments on LEDGF/p75 knockout cells supported these findings and revealed a significant percentage of HIV-1 proviruses aberrantly located near TSSs in the absence of the host factor LEDGF/p75[12,13]. Therefore, one of the most appealing opinion suggests the presence of alternative factor/s that could help the virus to integrate in absence of LEDGF/p75, as suggested by results obtained in LEDGF/p75 and HRP2 double KD cells, which show more than expected proviral integrations in active genes[14]. Based on previous notions and on our results we think that the nuclear basket components, in particular Tpr, could have a critical role in HIV integration sites selection, forming a link between viral nuclear translocation and integration. The role of Tpr in LEDGF/p75 and HRP2 double KD cells could explain the portion of proviruses integrated in active genes. This model is supported by the critical role of the nuclear basket in chromatin organization[15], which according to our data is essential for efficient viral replication. Importantly, previous studies reported that the absence of Tpr induces the presence of heterochromatin near the nuclear basket[15]. In Tpr depleted cells we observed a reduction of H3K36me3 underneath NPCs (**Fig.6**). Since LEDGF/p75 is directly associated with H3K36me3[6,7], Tpr depletion could provoke a displacement of LEDGF/p75, which could in turn explain

the loss of the usual viral chromatin targets. We and others previously showed that HIV-1 integration sites change when the integrity of the nuclear basket is perturbed by knocking down Nup153[2,3]. However, the depletion of Nup153 causes the concomitant loss of Tpr. In our study we were able to distinguish between the role of two nuclear basket Nups, Nup153 and Tpr, attributing a specific role to each one in the early steps of HIV life cycle (**Fig.1**) and showing that the main component of the nuclear basket, Tpr, has a critical role in HIV-1integration in active chromatin regions near the NPC.

These results corroborated the importance of the link between viral nuclear translocation and integration, showing that the disruption of this link promotes viral change in integration sites, even though the virus efficiently integrates in the host chromatin.

Supplementary Methods

Cell proliferation and Cell cycle

CellTiter 96® AQueous One Solution Cell Proliferation Assay which is a colorimetric method has been used for determining the number of viable cells in proliferation (**Supplementary Fig.1a**). Samples were analyzed at 490nm using a 96-well plate reader. Propidium Iodide Labelling and Flow Cytometry were performed on clones fixed in cold 1:1 ethanol (80%)/Acetone for 1 hr at -20°C. After clones were washed and resuspended in propidium iodide (10 mg/mL) and RNase (50 mg/ml) for 30 min at 37°C . Flow cytometry data was analysed using FlowJo (**Supplementary Fig.1b**)

RNA fractionation

To test the RNA export in clones and in Tpr KD bulk, RNA Nuclear and cytoplasmic were fractionated and extracted using the PARIS kit (Ambion) according the manufacturer's instructions. Nup214 KD bulk has been introduced as control. Cytoplasmic and nuclear RNA were quantified with a Nanodrop ND-1000, loaded on 1% agarose-formaldehyde gel and quantified by ImageJ (**Supplementary Fig.2**).

Image analysis

Image analysis was carried out using ImageJ and custom-made Matlab tools to quantify intranuclear intensities in almost 20 nuclei per condition (**Supplementary Fig.6b,c,d;**). Our MatLab tools allowed to outline the nuclear envelope based on the Hoechst image. The nuclear periphery was then defined as the inner region within 2 μm from this polygon. The fluorescence in the red channel was then integrated separately in the peripheral and central regions, and divided by the corresponding area.

Supplementary References

1 Ciuffi, A. *et al.* A role for LEDGF/p75 in targeting HIV DNA integration. *Nat Med* **11**, 1287-1289, doi:nm1329 [pii]10.1038/nm1329 (2005).

2 Koh, Y. *et al.* Differential effects of human immunodeficiency virus type 1 capsid and cellular factors nucleoporin 153 and LEDGF/p75 on the efficiency and specificity of viral DNA integration. *J Virol* **87**, 648-658, doi:10.1128/JVI.01148-12JVI.01148-12 [pii] (2013).

3 Di Nunzio, F. *et al.* Nup153 and Nup98 bind the HIV-1 core and contribute to the early steps of HIV-1 replication. *Virology* **440**, 8-18, doi:10.1016/j.virol.2013.02.008S0042-6822(13)00094-9 [pii] (2013).

4 Di Nunzio, F. New insights in the role of nucleoporins: a bridge leading to concerted steps from HIV-1 nuclear entry until integration. *Virus Res* **178**, 187-196, doi:10.1016/j.virusres.2013.09.003S0168-1702(13)00295-5 [pii] (2013).

5 Kvaratskhelia, M., Sharma, A., Larue, R. C., Serrao, E. & Engelman, A. Molecular mechanisms of retroviral integration site selection. *Nucleic Acids Res* **42**, 10209-10225, doi:10.1093/nar/gku769gku769 [pii] (2014).

6 Pradeepa, M. M., Sutherland, H. G., Ule, J., Grimes, G. R. & Bickmore, W. A. Psip1/Ledgf p52 binds methylated histone H3K36 and splicing factors and contributes to the regulation of alternative splicing. *PLoS Genet* **8**, e1002717, doi:10.1371/journal.pgen.1002717PGENETICS-D-11-02810 [pii] (2012).

7 Eidahl, J. O. *et al.* Structural basis for high-affinity binding of LEDGF PWWP to mononucleosomes. *Nucleic Acids Res* **41**, 3924-3936, doi:10.1093/nar/gkt074gkt074 [pii] (2013).

8 Gijsbers, R. *et al.* LEDGF hybrids efficiently retarget lentiviral integration into heterochromatin. *Mol Ther* **18**, 552-560, doi:10.1038/mt.2010.36mt201036 [pii] (2010).

9 Ferris, A. L. *et al.* Lens epithelium-derived growth factor fusion proteins redirect HIV-1 DNA integration. *Proc Natl Acad Sci U S A* **107**, 3135-3140, doi:10.1073/pnas.09141421070914142107 [pii] (2010).

10 Silvers, R. M. *et al.* Modification of integration site preferences of an HIV-1-based vector by expression of a novel synthetic protein. *Hum Gene Ther* **21**, 337-349, doi:10.1089/hum.2009.134 (2010).

11 Wang, H. *et al.* Efficient Transduction of LEDGF/p75 Mutant Cells by Gain-of-Function HIV-1 Integrase Mutant Viruses. *Mol Ther Methods Clin Dev* **1**, doi:10.1038/mtm.2013.2 (2014).

12 Marshall, H. M. *et al.* Role of PSIP1/LEDGF/p75 in lentiviral infectivity and integration targeting. *PLoS One* **2**, e1340, doi:10.1371/journal.pone.0001340 (2007).

13 Shun, M. C. *et al.* LEDGF/p75 functions downstream from preintegration complex formation to effect gene-specific HIV-1 integration. *Genes Dev* **21**, 1767-1778, doi:21/14/1767 [pii]10.1101/gad.1565107 (2007).

14 Wang, H. *et al.* HRP2 determines the efficiency and specificity of HIV-1 integration in LEDGF/p75 knockout cells but does not contribute to the antiviral activity of a potent LEDGF/p75-binding

site integrase inhibitor. *Nucleic Acids Res* **40**, 11518-11530, doi:10.1093/nar/gks913gks913 [pii] (2012).

15 Krull, S. *et al.* Protein Tpr is required for establishing nuclear pore-associated zones of heterochromatin exclusion. *EMBO J* **29**, 1659-1673, doi:10.1038/emboj.2010.54emboj201054 [pii] (2010).
